# Supplementary material for: Intimate partner violence by men living with HIV in Cameroon: Prevalence, associated factors and implications for HIV transmission risk (ANRS-12288 EVOLCAM)
Source: PLoS One. 2021 Feb 18;16(2):e0246192. doi: 10.1371/journal.pone.0246192 (PMC7891744; doi:10.1371/journal.pone.0246192)
Supplement: S1 File — (PDF) [file pone.0246192.s001.pdf]

MODULE A : CARACTÉRISTIQUES SOCIODÉMOGRAPHIQUES ET ÉCONOMIQUES DU MÉNAGE

Q001 - Date de naissance *(jj/mm/aaaa)* J J / M M / A A A A

Q002 - Quelle est la classe la plus élevée que vous avez fréquentée avec succès, ou équivalent ?

On entend par classe suivie avec succès, une année scolaire sanctionnée par le passage dans la classe supérieure et/ou l'acquisition d'un diplôme. Cocher une seule case

Système francophone		Système anglophone	
☐ 01.	Jamais fréquenté l'école	01.	Never gone to school
☐ 02.	Maternelle/ SIL	02.	Nursery/ Class1/ Class 2
☐ 03.	CP/ CPS	03.	Class 3
☐ 04.	CE1	04.	Class 4
☐ 05.	CE2	05.	Class 5
☐ 06.	CM1	06.	Class 6
☐ 07.	CM2	07.	Class 7
☐ 08.	6ème G ou 1ère A.T	08.	Form 1
☐ 09.	5ème G ou 2ème A.T	09.	Form 2
☐ 10.	4ème G ou 3ème A.T	10.	Form 3
☐ 11.	3ème G ou 4ème A.T ou BEPC ou CAP	11.	Form 4
☐ 12.	2nde G ou T	12.	Form 5
☐ 13.	1ère G ou T	13.	Lower sixth
☐ 14.	Terminale G ou T ou Bac	14.	Upper sixth
☐ 15.	1ère année Univ./ IUT/ BTS	15.	1st year Univ.
☐ 16.	2ème année Univ./ IUT/ BTS	16.	2nd year Univ.
☐ 17.	3ème année Univ.	17.	3rd year Univ.
☐ 18.	4ème année Univ.	18.	4th year Univ.
☐ 19.	5ème année Univ. ou plus	19.	5th year Univ. or more
☐ 20.	Autre		
☐ 21.	Refus de répondre (ne pas citer)		

Précisez ________________________________

Ecrire en majuscule

Q003 - Où habitez-vous ? Ne pas citer. Pour Douala ou Yaoundé préciser le quartier. Ecrire en majuscule

☐ 1.Dans la ville où est situé cet hôpital	☐ 2.En dehors de la ville où est situé l'hôpital	☐ 3.Refus de répondre (ne pas citer)
**Q003_3 - Si Douala ou Yaoundé, précisez le quartier**<br>☐ 1.Ne sait pas (ne pas citer)<br>☐ 2.Refus de répondre (ne pas citer)	**Q003_1 - Précisez le département**<br>☐ 1.Ne sait pas (ne pas citer)<br>☐ 2.Refus de répondre (ne pas citer)<br>**Q003_2 - Précisez le nom de la ville/ village**<br>☐ 1.Ne sait pas (ne pas citer)<br>☐ 2.Refus de répondre (ne pas citer)<br>**Q003_3 - Si Douala ou Yaoundé, précisez le quartier**<br>☐ 1.Ne sait pas (ne pas citer)<br>☐ 2.Refus de répondre (ne pas citer)	→ **Passer à la question Q004**

Q004 - Combien d'adultes et d'enfants vivent dans votre foyer/ ménage, vous y compris ?

Q004_1 - Nombre d'adultes (18 ans ou plus) |__|__|
- ☐ 1.Ne sait pas (ne pas citer)
- ☐ 2.Refus de répondre (ne pas citer)

Q004_2 - Nombre d'enfants (moins de 18 ans) |__|__|
- ☐ 1.Ne sait pas (ne pas citer)
- ☐ 2.Refus de répondre (ne pas citer)

Q005 - Quel est votre lien de parenté avec le chef de ménage ? Une seule réponse possible

- ☐ 1.Vous êtes le chef de ménage → **Aller à Q006 (page suivante)**
- ☐ 2.Vous êtes sa femme (ou son mari)
- ☐ 3.Vous êtes son frère (ou sa sœur)
- ☐ 4.Vous avez un lien de parenté ascendant (son père, sa mère, son grand père, sa grand-mère, son oncle, sa tante)
- ☐ 5.Vous avez un lien de parenté descendant (son fils, sa fille, son petit fils, sa petite fille, son neveu, sa nièce)
- ☐ 6.Autre lien de parenté (son cousin, sa cousine)
- ☐ 7.Sans lien de parenté **Précisez** ______________________ Ecrire en majuscule
- ☐ 8.Refus de répondre (ne pas citer)

Si la personne interrogée n'est PAS le chef de ménage (Q005 > 1), répondre à l'encadré suivant :

Q005_1 - Quelle est actuellement la situation professionnelle du chef de ménage ? Une seule réponse possible

- ☐ 1.Actif, occupé(e) (il ou elle travaille/ a une activité économique) → **Répondre à l'encadré 1**
- ☐ 2.Retraité/ pensionnaire/ personne âgée
- ☐ 3.Au chômage, à la recherche d'un travail
- ☐ 4.En attente de reprendre le travail lorsque son état de santé sera amélioré

(2, 3, 4) → **Répondre à l'encadré 2**

- ☐ 5.Autre (Sans activité économique, femme/homme au foyer, étudiant) **Précisez** ______________ Ecrire en majuscule
- ☐ 6.Ne sait pas (ne pas citer)
- ☐ 7.Refus de répondre (ne pas citer)

(5, 6, 7) → **Aller à Q006 (page suivante)**

Encadré 1 : Si le chef de ménage est actif (Q005_1 = 1)

Q005_2 - Quelle est la principale activité du chef de ménage ?

Demander au patient de décrire ce que fait le chef de ménage comme activité. Ecrire en majuscule

____
____
____
____
____
____
____
____

- ☐ 1.Ne sait pas (ne pas citer)
- ☐ 2.Refus de répondre (ne pas citer)

Q005_3 - S'agit-il d'une activité régulière ou occasionnelle ?

- ☐ 1.Régulière
- ☐ 2.Occasionnelle
- ☐ 3.Ne sait pas (ne pas citer)
- ☐ 4.Refus de répondre (ne pas citer)

Encadré 2 : Si le chef de ménage est inactif (Q005_1 = 2, 3 ou 4)

Q005_4 - Quelle était la dernière activité du chef de ménage ?

Demander au patient de décrire ce que faisait le chef de ménage comme activité quand il travaillait. Ecrire en majuscule

____
____
____
____
____
____
____
____

- ☐ 1.Ne sait pas (ne pas citer)
- ☐ 2.Refus de répondre (ne pas citer)

Q005_5 - S'agissait-il d'une activité régulière ou occasionnelle ?

- ☐ 1.Régulière
- ☐ 2.Occasionnelle
- ☐ 3.Ne sait pas (ne pas citer)
- ☐ 4.Refus de répondre (ne pas citer)

Q006 - Parmi les membres de votre ménage, combien de personnes contribuent de façon régulière au revenu du foyer/ ménage, vous y compris ?

|__|__| personnes ☐ 1.Ne sait pas (ne pas citer) ☐ 2.Refus de répondre (ne pas citer)

Q007 - Au cours du mois précédent, quel a été le revenu de votre ménage (en tenant compte des contributions financières de toutes les personnes de votre ménage et de toutes les sources de revenu, y compris aide de la famille, pensions, location de terrain, etc.) ?

Si le patient répond "ça dépend des mois" et donne plusieurs montants, cocher "Ne sait pas" et énumérer l'échelle Q007_1

|__| |__|__|__| |__|__|__| FCFA/mois

☐ 1.Ne sait pas (ne pas citer) ☐ 2.Refus de répondre (ne pas citer)

En cas de réponse spontanée, aller à Q008 sans énumérer l'échelle

Q007_1 - Pourriez-vous indiquer à peu près quel a été le revenu mensuel de votre ménage au cours du mois précédent ?

Une seule réponse possible

- ☐ 01.Moins de 10 000 FCFA
- ☐ 02.Entre 10 000 et 30 000 FCFA
- ☐ 03.Entre 30 000 et 50 000 FCFA
- ☐ 04.Entre 50 000 et 70 000 FCFA
- ☐ 05.Entre 70 000 et 100 000 FCFA
- ☐ 06.Entre 100 000 et 150 000 FCFA
- ☐ 07.Entre 150 000 et 300 000 FCFA
- ☐ 08.Entre 300 000 et 500 000 FCFA
- ☐ 09.Plus de 500 000 FCFA
- ☐ 10.Ne sait pas (ne pas citer)
- ☐ 11.Refus de répondre (ne pas citer)

Q008 - Au cours du mois précédent, combien avez-vous dépensé environ dans votre ménage pour

Inscrire 0 si aucune dépense

		(ne pas citer) 1.Ne sait pas	2.Refus de répondre
L'alimentation (y.c. eau/ produits d'entretien/ hygiène)	|__| |__|__|__| |__|__|__| FCFA	☐	☐
Le logement (loyer + électricité + petits travaux d'entretien)	|__| |__|__|__| |__|__|__| FCFA	☐	☐
Les sorties/ loisirs (restaurant, bars, ….)/ voyage	|__| |__|__|__| |__|__|__| FCFA	☐	☐
Le transport (taxis, carburant, …)	|__| |__|__|__| |__|__|__| FCFA	☐	☐
Les communications (téléphone)	|__| |__|__|__| |__|__|__| FCFA	☐	☐

Q009 - Au cours des 6 mois précédents, combien avez-vous dépensé environ dans votre ménage pour

Inscrire 0 si aucune dépense

		(ne pas citer) 1.Ne sait pas	2.Refus de répondre
	|__| |__|__|__| |__|__|__| FCFA		
L'éducation (frais de scolarité, livres, cahiers, …)	|__| |__|__|__| |__|__|__| FCFA	☐	☐
Les vêtements/ coiffure/ soins de beauté	|__| |__|__|__| |__|__|__| FCFA	☐	☐
Les biens d'équipement de la maison (appareils électroménagers, meubles, ...)	|__| |__|__|__| |__|__|__| FCFA	☐	☐
Aider la famille/ des amis	|__| |__|__|__| |__|__|__| FCFA	☐	☐

Q010 - Votre ménage est-il

- ☐ 1.Propriétaire
- ☐ 2.Locataire (vous payez un loyer)
- ☐ 3.Autre **Précisez** ____________ Ecrire en majuscule
- ☐ 4.Ne sait pas (ne pas citer)
- ☐ 5.Refus de répondre (ne pas citer)

Q011 - Est-ce que votre logement dispose

Une réponse par ligne

	1.Oui	2.Non	(ne pas citer) 3.Refus de répondre
D'une salle d'eau (c'est-à-dire eau courante avec douche)	☐	☐	☐
De WC avec chasse d'eau	☐	☐	☐
De latrines traditionnelles	☐	☐	☐
D'un évier dans la cuisine	☐	☐	☐
De l'électricité	☐	☐	☐
De la climatisation	☐	☐	☐
D'un réfrigérateur	☐	☐	☐
D'une cuisinière à gaz ou électrique avec four	☐	☐	☐
D'une connexion à internet	☐	☐	☐

Q012 - Quel est le principal matériau de construction des murs de votre maison ?

Ecrire en majuscule

- ☐ 1.Ciment/ briques
- ☐ 2.Planches/ caraboat
- ☐ 3.Tôle
- ☐ 4.Briques de terre
- ☐ 5.Autre **Précisez** ____________
- ☐ 6.Ne sait pas (ne pas citer)
- ☐ 7.Refus de répondre (ne pas citer)

Q013 - Votre ménage possède-t-il les biens suivants ?

Une réponse par ligne

	1.Oui	2.Non	(ne pas citer) 3.Ne sait pas	(ne pas citer) 4.Refus de répondre
Voiture, camionnette	☐	☐	☐	☐
Télévision	☐	☐	☐	☐
Radio	☐	☐	☐	☐
Ordinateur	☐	☐	☐	☐
Un ou des terrains agricoles	☐	☐	☐	☐

Q014 - Au cours des 4 dernières semaines, est-ce que vous avez mangé au moins deux fois par jour ?

Une seule réponse possible

- ☐ 1.Toujours
- ☐ 2.Presque toujours (en moyenne 5 à 6 jours sur 7)
- ☐ 3.Parfois (en moyenne 3 à 4 jours sur 7)
- ☐ 4.Rarement (moins de 2 fois par semaine)
- ☐ 5.Jamais
- ☐ 6.Ne sait pas (ne pas citer)
- ☐ 7.Refus de répondre (ne pas citer)

MODULE B : ACTIVITÉ PROFESSIONNELLE DU PATIENT

A présent je vais vous poser des questions au sujet de votre activité professionnelle.

Q015 - Actuellement, travaillez-vous ou avez-vous une activité qui vous permet de gagner de l'argent, même si c'est peu ?

On considère comme travail toutes les activités qui permettent de gagner de l'argent, comme le travail aux champs (même s'il n'est pas payé, il permet d'avoir un revenu grâce à la vente des produits). Les activités ménagères à la maison ne sont pas considérées comme un travail

☐ 1.Oui → ☐ 2.Non, pas du tout → ☐ 3.Refus de répondre (ne pas citer)

Si Oui (Q015 = 1), répondre à l'encadré ci-dessous :

Q015_1 - Actuellement, quelle est votre activité principale ?

Demander au patient de décrire ce qu'il fait. Ecrire en majuscule

____

☐ 1.Ne sait pas (ne pas citer) ☐ 2.Refus (ne pas citer)

Q015_2 - S'agit-il d'une activité régulière ou occasionnelle ?

☐ 1.Régulière ☐ 2.Occasionnelle

☐ 3.Refus de répondre (ne pas citer)

Q015_3 - Combien de personnes travaillent avec vous ?

☐ 1.Entre 1 et 10 ☐ 2.Plus de 10

☐ 3.Refus de répondre (ne pas citer)

Q015_4 - Vous travaillez comme Une seule réponse possible

☐ 1.Fonctionnaire

☐ 2.Salarié (quel que soit le secteur, y.c. salarié chez un particulier)

☐ 3.Indépendant ou à votre compte (sans employés à votre charge)

☐ 4.Chef d'entreprise (au moins un employé à charge)

☐ 5.Apprenti ou aide familiale non salarié

☐ 6.Autre **Précisez** ______________________ Ecrire en majuscule

☐ 7.Refus de répondre (ne pas citer)

<u>Au cours du mois précédent :</u>

Q015_5 - Combien avez-vous gagné ?

|_| |_|_|_| |_|_|_| FCFA

☐ 1.Ne sait pas (ne pas citer)

☐ 2.Refus de répondre (ne pas citer)

Q015_6 - Combien de jours environ avez-vous travaillé ?

|_|_| jours

☐ 1.Ne sait pas (ne pas citer)

☐ 2.Refus de répondre (ne pas citer)

Q015_7 - Lorsque vous avez travaillé, combien d'heures environ par jour avez-vous travaillé ?

|_|_| heures/jour

☐ 1.Ne sait pas (ne pas citer)

☐ 2.Refus de répondre (ne pas citer)

Q015_8 - Vous est-il arrivé de ne pas pouvoir travailler parce que vous étiez malade ?

☐ 1.Oui → **Combien de jours ?** |_|_| jours

☐ Ne sait pas combien (ne pas citer)

☐ 2.Non

☐ 3.Refus de répondre (ne pas citer)

Si Non ou Refus (Q015 = 2 ou 3), répondre à l'encadré ci-dessous :

Q015_9 - Quelle est votre situation actuelle ? Une seule réponse possible

☐ 1.Etudiant

☐ 2.Femme au foyer (ménagère) ou femme enceinte/ post grossesse

☐ 3.Retraité/ pensionnaire/ personne âgée

☐ 4.Au chômage ou à la recherche d'un travail

☐ 5.En attente de reprendre le travail lorsque votre état de santé sera amélioré

☐ 6.Invalidité/ handicap permanent

☐ 7.Autre **Précisez** ______________________ Ecrire en majuscule

☐ 8.Refus de répondre (ne pas citer)

Q016 - Au cours du mois précédent, est-ce que quelqu'un vous a aidé pour des tâches/ activités de la vie quotidienne (préparation des repas, garde des enfants, toilette, marche, ...) parce que vous étiez malade ?

☐ 1.Oui ☐ 2.Non ☐ 3.Refus de répondre (ne pas citer)

Passer à la question Q017 (page suivante)

Si Oui (Q016 = 1) répondre à l'encadré ci-dessous :

Q016_1 - Concernant la personne qui vous a aidé (aide principale si plusieurs) au cours du mois précédent :

Q016_1_1 - Cette personne vit-elle dans votre ménage/ foyer ?

☐ 1.Oui
☐ 2.Non
☐ 3.Refus de répondre (ne pas citer)

Q016_1_2 - Quel est environ l'âge de la personne qui vous a aidé ?

|__|__| ans

☐ 1.Ne sait pas (ne pas citer)
☐ 2.Refus de répondre (ne pas citer)

Q016_1_3 - Payez-vous cette personne pour l'aide qu'elle vous donne ?

☐ 1.Oui
☐ 2.Non
☐ 3.Refus de répondre (ne pas citer)

Q016_2 - Au cours du mois précédent, combien de jours avez-vous eu besoin de l'aide de cette personne ?

|__|__| jours ☐ 1.Ne sait pas (ne pas citer) ☐ 2.Refus de répondre (ne pas citer)

Q016_3 - La personne qui vous a aidé au cours du mois précédent a-t-elle dû arrêter (complètement ou partiellement) son travail habituel (payé ou non) ?

☐ 1.Oui
☐ 2.Non
☐ 3.Non concerné (la personne ne travaille pas)
☐ 4.Ne sait pas (ne pas citer)
☐ 5.Refus de répondre (ne pas citer)

MODULE C : HISTOIRE ET TRAITEMENT DE L'INFECTION À VIH

Nous allons maintenant parler des problèmes de santé, des soins et des traitements que vous avez reçus en commençant par le moment où vous avez appris votre séropositivité.

Q017 - Quand avez-vous appris que votre test pour le VIH était positif ?

Demander la date précise ; inscrire la durée approximative si le patient n'a pas souvenir de la date exacte

Mois |M|M| **Année** |A|A|A|A| **OU** **Nombre de mois** |__|__| **Nombre d'années** |__|__|

☐ 1.Ne sait pas (ne pas citer) ☐ 2.Refus de répondre (ne pas citer)

Q018 - Avez-vous fait ce test Une seule réponse possible

☐ 1.De vous-même
☐ 2.A la demande d'un personnel soignant
☐ 3.A la demande de votre partenaire
☐ 4.Le test a été fait sans que vous le sachiez
☐ 5.Refus de répondre (ne pas citer)

Q019 - Dans quelle circonstance avez-vous fait ce test ? Une seule réponse possible

☐ 1.Suite à des symptômes pour une maladie (fièvre, amaigrissement...)
☐ 2.A l'occasion d'un suivi de grossesse (consultation prénatale) ou d'un accouchement
☐ 3.A l'occasion d'une campagne de dépistage
☐ 4.Suite à la découverte de l'infection d'une personne de votre entourage **Précisez qui** ____________
☐ 5.Autre (bilan médical) **Précisez** ____________ Ecrire en majuscule
☐ 6.Refus de répondre (ne pas citer)

Q020 - Après avoir appris le résultat positif de votre test, combien de temps s'est écoulé avant que vous alliez consulter un personnel soignant pour le suivi de votre infection à VIH ?

☐ 1.Moins d'un mois
☐ 2.Entre 1 et 3 mois
☐ 3.Entre 3 et 6 mois
☐ 4.Entre 6 mois et un an
☐ 5.Plus d'un an
☐ 6.Refus de répondre (ne pas citer)

Nous allons parler maintenant du traitement contre l'infection à VIH qu'on appelle traitement antirétroviral ou ARV.

Q021 - Pensez-vous que le traitement ARV

NSP = Ne sait pas (ne pas citer)

Une réponse par ligne	1.Pas du tout	2.Un peu	3.Assez	4.Beaucoup	5.NSP	6.Refus
Réduit le risque de transmission du VIH pendant les rapports sexuels	☐	☐	☐	☐	☐	☐
Réduit le risque de transmission du VIH au bébé pendant la grossesse	☐	☐	☐	☐	☐	☐

TRAITEMENTS ET SUIVI MÉDICAL

Q022 - En général, par rapport aux ordonnances médicales (tous traitements confondus) vous diriez que vous les suivez, sur une échelle de 1 à 6

Entourer le chiffre indiqué par le patient

1 (jamais) — (toujours) 6

1 2 3 4 5 6

☐ 1.Ne sait pas (ne pas citer) ☐ 2.Refus de répondre (ne pas citer)

Q023 - Au cours du dernier mois, avez-vous pris un traitement ARV ? ☐ Refus de répondre (ne pas citer)

☐ 1.Oui	☐ 2.Non, mais vous avez déjà reçu un traitement dans le passé	☐ 3.Non, vous n'avez jamais reçu de traitement
**Q023_1 - Quand avez-vous commencé à prendre ce traitement ?**<br>Mois |M|M| Année |A|A|A|A| ☐ 1.Ne sait pas (ne pas citer)<br>☐ 2.Refus de répondre (ne pas citer)<br><br>**Q023_2 - Au cours des 12 derniers mois, avez-vous eu un entretien individuel avec un conseiller pour discuter de ce traitement ?**<br>Le conseiller est différent du médecin. Il peut être infirmier, assistant social, psychologue<br>☐ 1.Oui, une fois<br>☐ 2.Oui, plusieurs fois<br>☐ 3.Non<br>☐ 4.Refus de répondre (ne pas citer)<br><br>→ **Passer à la section OBSERVANCE page suivante (Q024)**	**Q023_3 - Combien de temps avez-vous suivi ce traitement avant de l'interrompre ?**<br>Nombre de mois |__|__| Nombre d'années |__|__| ☐ 1.Ne sait pas (ne pas citer)<br>☐ 2.Refus de répondre (ne pas citer)<br><br>**Q023_4 - Depuis combien de temps l'avez-vous arrêté ?**<br>☐ 1.Moins de 3 mois<br>☐ 2.Entre 3 et 6 mois<br>☐ 3.Plus de 6 mois<br>☐ 4.Refus de répondre (ne pas citer)<br><br>→ **Passer au module D page 14**	**Q023_5 - Actuellement, attendez-vous le résultat de vos examens ou un rendez-vous avec le médecin pour démarrer bientôt un traitement ?**<br>☐ 1.Oui<br>☐ 2.Non<br>☐ 3.Refus de répondre (ne pas citer)<br><br>→ **Passer au module D page 14**

OBSERVANCE

Q024 - Maintenant, nous allons nous intéresser au traitement ARV que vous avez pris ces 4 derniers jours, en commençant à partir d'hier. Utiliser les images de médicaments en couleur pour identifier les médicaments avec le patient

	Quel est le nom de votre (vos) médicament(s) ARV contre le VIH ? Ecrire en majuscule	Quel est le nombre de comprimés prescrits par jour par le médecin ?	Combien de comprimés avez-vous pris			
			Hier ?	Avant-hier ?	Il y a 3 jours ?	Il y a 4 jours ?
	Exemple : TRIOMUNE 30	*Exemple : 2 comprimés / jour*	*Exemple : 2*	*Exemple : 0*	*Exemple : 1*	*Exemple : 0*
**Médicament 1**						
**Médicament 2**						
**Médicament 3**						
**Médicament 4**						
**Médicament 5**						

Q025 - Au cours de ces 4 derniers jours, vous est-il arrivé de prendre la dose journalière de votre (vos) médicament(s) ARV en une seule fois ?

- ☐ 1.Oui, toujours car mon traitement se prend en une seule fois par jour
- ☐ 2.Oui, plusieurs fois
- ☐ 3.Oui, une fois
- ☐ 4.Non, jamais
- ☐ 5.Refus de répondre (ne pas citer)

Q026 - Au cours de ces 4 derniers jours, vous est-il arrivé d'oublier ou de décaler de plusieurs heures, l'heure de la prise de votre (vos) médicament(s) ARV ?

- ☐ 1.Oui, plusieurs fois
- ☐ 2.Oui, une fois
- ☐ 3.Non, jamais
- ☐ 4.Refus de répondre (ne pas citer)

Q027 - Au cours de ces 4 derniers jours, concernant votre traitement ARV, avez-vous Une seule réponse possible

- ☐ 1.Interrompu tout traitement à la demande du médecin
- ☐ 2.Interrompu tout traitement pour des raisons personnelles
- ☐ 3.Continué à le suivre partiellement
- ☐ 4.Continué à le suivre totalement
- ☐ 5.Refus de répondre (ne pas citer)

Q028 - On observe qu'il est souvent plus difficile pour les gens de prendre leur traitement durant le week-end (samedi-dimanche). En ce qui vous concerne, avez-vous manqué un ou plusieurs comprimés de votre traitement ARV contre le VIH lors du dernier week-end ?

- ☐ 1.Oui
- ☐ 2.Non
- ☐ 3.Refus de répondre (ne pas citer)

Nous allons maintenant nous intéresser au traitement ARV contre le VIH tel que vous l'avez pris ces 4 dernières semaines.

Q029 - Selon vous, au cours des 4 dernières semaines, vous avez Une seule réponse possible

- ☐ 1.Scrupuleusement respecté les prescriptions médicales
- ☐ 2.Globalement respecté les prescriptions médicales avec quelques écarts
- ☐ 3.Souvent modifié les prises (rythmes et quantités)
- ☐ 4.Pratiquement jamais respecté les prescriptions médicales
- ☐ 5.Arrêté tout traitement
- ☐ 6.Refus de répondre (ne pas citer)

Q030 - Au cours des 4 dernières semaines, avez-vous interrompu votre traitement pendant plus de 2 jours ?

- ☐ 1.Oui, plusieurs fois
- ☐ 2.Oui, une fois
- ☐ 3.Non, jamais
- ☐ 4.Refus de répondre (ne pas citer)

Q031 - Au cours des 3 derniers mois, vous est-il arrivé de ne pas trouver un ou plusieurs médicaments de votre traitement à la pharmacie de l'hôpital où vous allez habituellement ?

- ☐ 1.Oui, au moins une fois → **Poser la question Q031_1**
- ☐ 2.Non, jamais → **Passer à la question Q032**
- ☐ 3.Refus de répondre (ne pas citer) → **Passer à la question Q032**

Q031_1 - Etait-ce dans les 4 dernières semaines ?

- ☐ 1.Oui
- ☐ 2.Non
- ☐ 3.Refus de répondre (ne pas citer)

Q032 - Au cours des 3 derniers mois, vous est-il arrivé de payer pour vous procurer votre traitement ARV ?

- ☐ 1.Oui, plusieurs fois
- ☐ 2.Oui, une fois
- ☐ 3.Non, jamais
- ☐ 4.Ne sait pas (ne pas citer)
- ☐ 5.Refus de répondre (ne pas citer)

Si Oui, plusieurs fois ou une fois (Q032 = 1 ou 2) répondre à l'encadré ci-dessous :

Q032_1 - Où avez-vous acheté ces ARV ?

Une réponse par ligne

	1.Oui	2.Non	3.Ne sait pas	4.Refus de répondre
Dans cet hôpital	☐	☐	☐	☐
Dans un autre hôpital, dispensaire ou centre de santé	☐	☐	☐	☐
Dans une pharmacie	☐	☐	☐	☐
Dans la rue ou sur le marché	☐	☐	☐	☐
Autre **Précisez** ______	☐	☐	☐	☐

Ecrire en majuscule

Q033 - Au cours des 3 derniers mois, vous est-il arrivé de ne pas pouvoir vous procurer votre traitement ARV faute d'argent ?

☐ 1.Oui, plusieurs fois
☐ 2.Oui, une fois
☐ 3.Non, jamais
☐ 4.Refus de répondre (ne pas citer)

Q034 - Depuis que vous avez commencé à prendre un traitement ARV, avez-vous déjà arrêté ce traitement pendant plus d'un mois ?

☐ 1.Oui, au moins une fois → **Poser les questions Q034_1 et Q034_2**
☐ 2.Non, jamais
☐ 3.Refus de répondre (ne pas citer)
→ **Passer au module D (page suivante)**

Q034_1 - Pendant combien de temps avez-vous arrêté votre traitement ?

☐ 1.Entre 1 et 3 mois
☐ 2.Entre 3 mois et un an
☐ 3.Plus d'un an
☐ 4.Ne sait pas (ne pas citer)
☐ 5.Refus de répondre (ne pas citer)

Q034_2 - Etait-ce dans les 6 derniers mois ?

☐ 1.Oui
☐ 2.Non
☐ 3.Ne sait pas (ne pas citer)
☐ 4.Refus de répondre (ne pas citer)

MODULE G : COMPORTEMENTS À RISQUE ET COMPORTEMENTS SEXUELS

Nous allons aborder maintenant des questions plus personnelles. Nous allons commencer par des questions concernant votre consommation d'alcool et d'autres produits que celle-ci soit occasionnelle ou plus régulière, puis nous aborderons des questions portant sur la sexualité.

CONSOMMATION D'ALCOOL ET AUTRES PRODUITS

Q072 - Buvez-vous de la bière ?

- ☐ 1.Jamais ➡ **Passer à la question Q073**
- ☐ 2.Une fois par mois ou moins
- ☐ 3.Entre 2 à 4 fois par mois
- ☐ 4.Entre 2 à 3 fois par semaine
- ☐ 5.Entre 4 à 6 fois par semaine
- ☐ 6.Tous les jours
- ☐ 7.Refus de répondre (ne pas citer) ➡ **Passer à la question Q073**

Q072_1 - Lorsque vous buvez de la bière, combien de grandes bouteilles (65 centilitres) ou de petites bouteilles (33cl) buvez-vous à peu près à chaque fois ?

- ☐ 1.Une grande bouteille de 65 cl ou moins (= moins de 2 petites bouteilles)
- ☐ 2.Entre 2 et 3 grandes bouteilles de 65 cl (= entre 3 et 6 petites bouteilles)
- ☐ 3.Entre 4 et 5 grandes bouteilles de 65 cl (= entre 7 et 10 petites bouteilles)
- ☐ 4.Plus de 5 grandes bouteilles de 65 cl (= plus de 11 petites bouteilles)
- ☐ 5.Ne sait pas (ne pas citer)
- ☐ 6.Refus de répondre (ne pas citer)

Q073 - Vous arrive-t-il de boire plus de trois grandes bouteilles de bière et/ ou plus de 6 verres (ou sachets) d'autres boissons alcoolisées, au cours d'une même occasion ?

- ☐ 1.Jamais
- ☐ 2.Moins d'une fois par mois
- ☐ 3.Une fois par mois
- ☐ 4.Une fois par semaine
- ☐ 5.Tous les jours ou presque
- ☐ 6.Non concerné (ne pas citer)
- ☐ 7.Ne sait pas (ne pas citer)
- ☐ 8.Refus de répondre (ne pas citer)

Q074 - Avez-vous déjà consommé du banga/ ndjap/ cannabis/ tramol/ caillou/ cocaine ne serait-ce qu'une seule fois au cours de votre vie, même il y a longtemps ?

- ☐ 1.Oui, au cours des 4 dernières semaines
- ☐ 2.Oui, au moins une fois dans votre vie
- ☐ 3.Non
- ☐ 4.Refus de répondre (ne pas citer)

SEXUALITÉ

Nous allons passer maintenant aux questions concernant votre sexualité.

Q075 - Au cours de votre vie, avec combien de partenaires différent(e)s avez-vous eu des relations sexuelles (y compris votre conjoint(e) actuel(le)) ? Demander le nombre de partenaires sexuels hommes ET le nombre de partenaires sexuels femmes au patient

Q075_1 - Nombre d'hommes

- ☐ 1.Aucun
- ☐ 2.Un
- ☐ 3.Entre 2 et 5
- ☐ 4.Entre 6 et 10
- ☐ 5.Entre 11 et 20
- ☐ 6.Entre 21 et 50
- ☐ 7.Plus de 50
- ☐ 8.Ne sait pas (ne pas citer)
- ☐ 9.Refus de répondre (ne pas citer)

Q075_2 - Nombre de femmes

- ☐ 1.Aucune
- ☐ 2.Une
- ☐ 3.Entre 2 et 5
- ☐ 4.Entre 6 et 10
- ☐ 5.Entre 11 et 20
- ☐ 6.Entre 21 et 50
- ☐ 7.Plus de 50
- ☐ 8.Ne sait pas (ne pas citer)
- ☐ 9.Refus de répondre (ne pas citer)

Q076 - A quel âge avez-vous eu ce que vous considérez comme votre premier rapport sexuel ?

|__|__| ans ☐ 1.Ne sait pas (ne pas citer) ☐ 2.Refus de répondre (ne pas citer)

Q077 - Aviez-vous utilisé un préservatif ?

- ☐ 1.Oui
- ☐ 2.Non
- ☐ 3.Ne sait pas (ne pas citer)
- ☐ 4.Refus de répondre (ne pas citer)

Q078 - Avez-vous un conjoint/ petit ami/ partenaire principal en ce moment ? ☐ Refus de répondre (ne pas citer)

☐ 1.Oui	☐ 2.Non
**Q078_1 - Êtes-vous**<br>☐ 1.Marié(e) légalement ou "coutumièrement"<br>☐ 2.En union libre ou consensuelle<br>☐ 3.Refus de répondre (ne pas citer)<br><br>**Q078_2 - Êtes-vous dans un mariage/ union polygame ?**<br>☐ 1.Oui<br>☐ 2.Non<br>☐ 3.Refus de répondre (ne pas citer)<br><br>**Q078_3 - Vivez-vous avec votre partenaire principal(e)/ conjoint(e) dans le même foyer ?**<br>☐ 1.Oui<br>☐ 2.Non<br>☐ 3.Refus de répondre (ne pas citer)<br><br>**Q078_4 - Depuis quand êtes-vous ensemble ?**<br>|__|__| mois **OU** |__|__| années<br>☐ 1.Ne sait pas (ne pas citer)<br>☐ 2.Refus de répondre (ne pas citer)<br><br>**Q078_5 - Quel est le niveau d'études de votre conjoint(e)/ partenaire ?**<br>☐ 1.N'est jamais allé(e) à l'école<br>☐ 2.Primaire (CP au CM2/ Class 3 à Class 7)<br>☐ 3.Secondaire 1er cycle (6ème à 3ème/ Form1 à Form4)<br>☐ 4.Secondaire 2ème cycle (2nde, 1ère, Terminale/ Form 5, Lower Sixth, Upper Sixth)<br>☐ 5.Université ou études supérieures après le bac<br>☐ 6.Ne sait pas (ne pas citer)<br>☐ 7.Refus de répondre (ne pas citer)	**Q078_6 - Êtes-vous**<br>☐ 1.Veuf(ve)<br>☐ 2.Célibataire → **Poser la question Q078_7**<br>☐ 3.Divorcé(e)/ séparé(e) → **Poser la question Q078_8**<br>☐ 4.Refus de répondre (ne pas citer) → **Passer à la question Q079**<br><br>**Q078_7 - Au cours des 12 derniers mois, avez-vous eu un partenaire principal avec qui vous avez rompu ?**<br>☐ 1.Oui → **Poser la question Q078_8**<br>☐ 2.Non<br>☐ 3.Refus de répondre (ne pas citer) → **Passer à la question Q079**<br><br>**Q078_8 - Votre séparation était-elle en relation avec votre statut VIH ?**<br>☐ 1.Oui<br>☐ 2.Non<br>☐ 3.Ne sait pas (ne pas citer)<br>☐ 4.Refus de répondre (ne pas citer)

Q079 - Au cours des 12 derniers mois, combien de partenaires sexuels avez-vous eu ?

\|__\|__\| partenaires sexuels

En cas de réponse spontanée, ne pas énumérer l'échelle

Si le patient ne donne pas de chiffre précis, énumérer l'échelle suivante

☐ 1.Aucun partenaire
☐ 2.Un partenaire
☐ 3.De 2 à 5 partenaires
☐ 4.De 6 à 10 partenaires
☐ 5.Plus de 10 partenaires
☐ 6.Ne sait pas
☐ 7.Refus de répondre
(ne pas citer)

Si le patient a eu au moins un partenaire (Q079 > 0) passer à la question Q080 (page suivante).

Si le patient n'a eu aucun partenaire sexuel au cours des 12 derniers mois (Q079 = 0) ou n'a pas répondu à la question Q079, passer au module sur les violences :
- → **Si le patient est un homme, aller page 28 à la question Q088_H**
- → **Si le patient est une femme, aller page 30 à la question Q088_F**

Je vais maintenant vous poser des questions concernant vos partenaires sexuels au cours des 12 derniers mois.

Poser les questions jusqu'à 2 partenaires, en commençant par le plus récent
Ne pas citer les mentions "Ne sait pas" et "Refus de répondre"

	Partenaire sexuel le plus récent	Partenaire sexuel précédent
**Q080 - Votre partenaire sexuel était**	☐ 1.Partenaire principal<br>☐ 2.Partenaire occasionnel<br>☐ 3.Refus de répondre	☐ 1.Partenaire principal<br>☐ 2.Partenaire occasionnel<br>☐ 3.Refus de répondre
**Q081 - Quel âge a-t-il ?**	☐ 1.Environ le même âge que vous (plus ou moins 5 ans)<br>☐ 2.Plus jeune que vous (écart d'âge supérieur à 5 ans)<br>☐ 3.Plus vieux que vous (écart d'âge supérieur à 5 ans)<br>☐ 4.Ne sait pas<br>☐ 5.Refus de répondre	☐ 1.Environ le même âge que vous (plus ou moins 5 ans)<br>☐ 2.Plus jeune que vous (écart d'âge supérieur à 5 ans)<br>☐ 3.Plus vieux que vous (écart d'âge supérieur à 5 ans)<br>☐ 4.Ne sait pas<br>☐ 5.Refus de répondre
**Q082 - Connaissez-vous son statut VIH ?**	☐ 1.Oui, séropositif<br>☐ 2.Oui, séronégatif<br>☐ 3.Ne sait pas<br>☐ 4.Refus de répondre	☐ 1.Oui, séropositif<br>☐ 2.Oui, séronégatif<br>☐ 3.Ne sait pas<br>☐ 4.Refus de répondre
**Q083 - Lui avez-vous révélé votre statut VIH ?**	☐ 1.Oui<br>☐ 2.Non<br>☐ 3.Ne sait pas<br>☐ 4.Refus de répondre	☐ 1.Oui<br>☐ 2.Non<br>☐ 3.Ne sait pas<br>☐ 4.Refus de répondre
**Q084 - Diriez-vous qu'en moyenne, avec ce partenaire, vous avez eu des relations sexuelles**	☐ 1.Moins d'une fois par mois<br>☐ 2.Une fois par mois<br>☐ 3.Plus d'une fois par mois<br>☐ 4.Plusieurs fois par semaine<br>☐ 5.Tous les jours<br>☐ 6.Ne sait pas<br>☐ 7.Refus de répondre	☐ 1.Moins d'une fois par mois<br>☐ 2.Une fois par mois<br>☐ 3.Plus d'une fois par mois<br>☐ 4.Plusieurs fois par semaine<br>☐ 5.Tous les jours<br>☐ 6.Ne sait pas<br>☐ 7.Refus de répondre
**Q085 - Lors de vos relations sexuelles avec ce partenaire, avez-vous utilisé des préservatifs**	☐ 1.Jamais<br>☐ 2.De temps en temps<br>☐ 3.Presque toujours<br>☐ 4.Toujours<br>☐ 5.Ne sait pas<br>☐ 6.Refus de répondre	☐ 1.Jamais<br>☐ 2.De temps en temps<br>☐ 3.Presque toujours<br>☐ 4.Toujours<br>☐ 5.Ne sait pas<br>☐ 6.Refus de répondre
**Q086 - Lors de votre dernière relation sexuelle avec ce partenaire, avez-vous utilisé un préservatif ?**	☐ 1.Oui<br>☐ 2.Non<br>☐ 3.Ne sait pas<br>☐ 4.Refus de répondre	☐ 1.Oui<br>☐ 2.Non<br>☐ 3.Ne sait pas<br>☐ 4.Refus de répondre

Q087 - Au cours des 12 derniers mois, avez-vous eu des rapports sexuels en payant et/ ou en étant payé ?

☐ 1.En payant
☐ 2.En étant payé ou en échange de cadeaux
☐ 3.Non
☐ 4.Ne sait pas (ne pas citer)
☐ 5.Refus de répondre (ne pas citer)

VIOLENCE CONJUGALE ET SEXUELLE

A NE POSER QUE SI LE PATIENT EST UN HOMME

Si le patient est une femme passer directement à la question Q088_F (page 30)

Je vais maintenant vous poser quelques questions personnelles concernant la paternité, le désir d'enfant, et enfin, les relations au sein du couple. Je vous rappelle que vos réponses sont confidentielles et anonymes et que je suis tenu(e) au secret professionnel.

Q088_H - Avez-vous des enfants ?

☐ 1.Oui → **Combien ?** |__|__|
☐ 1.Ne sait pas (ne pas citer)
☐ 2.Refus de répondre (ne pas citer)

☐ 2.Non
☐ 3.Refus de répondre (ne pas citer)

Q089 - Actuellement, souhaitez-vous ou essayez-vous d'avoir un enfant avec votre femme/ partenaire ?

☐ 1.Oui
☐ 2.Non
☐ 3.Non concerné (homme âgé etc.)
☐ 4.Ne sait pas (ne pas citer)
☐ 5.Refus de répondre (ne pas citer)

Q090 - Êtes-vous circoncis ?

☐ 1.Oui, de façon traditionnelle
☐ 2.Oui, de façon médicale
☐ 3.Non
☐ 4.Refus de répondre (ne pas citer)

Maintenant, si vous le permettez, je vais vous poser des questions concernant vos relations avec votre partenaire actuel(le) ou votre dernier(e) partenaire si vous n'avez pas de partenaire en ce moment.

Si le patient répond spontanément qu'il n'a jamais eu de partenaire, cocher la case "N'a jamais eu de partenaire" et passer directement au module H (page 32). **N'a jamais eu de partenaire** ☐

Q092 - Qui décide habituellement comment l'argent que vous gagnez va être utilisé ?

Ne pas citer les modalités, laissez la personne répondre spontanément

☐ 1.L'enquêté
☐ 2.La femme/ partenaire
☐ 3.L'enquêté et femme/ partenaire ensemble
☐ 4.Quelqu'un d'autre
☐ 5.L'enquêté et quelqu'un d'autre ensemble
☐ 6.Autre **Précisez** ________________ **Ecrire en majuscule**
☐ 7.Refus de répondre (ne pas citer)

Q093 - Qui habituellement a le dernier mot dans les décisions concernant vos propres soins de santé ?

Ne pas citer les modalités, laissez la personne répondre spontanément

☐ 1.L'enquêté
☐ 2.La femme/ partenaire
☐ 3.L'enquêté et femme/ partenaire ensemble
☐ 4.Quelqu'un d'autre
☐ 5.L'enquêté et quelqu'un d'autre ensemble
☐ 6.Autre **Précisez** ________________ **Ecrire en majuscule**
☐ 7.Refus de répondre (ne pas citer)

Ne pas citer les mentions "Refus"

Q094 - Avez-vous déjà		Au cours des 12 derniers mois, cela est-il arrivé :
Dit ou fait quelque chose pour humilier votre (dernière) partenaire devant d'autres personnes ?	☐ 1.Oui → ☐ 2.Non	☐ 1.Souvent ☐ 2.Parfois ☐ 3.Jamais ☐ 4.Non concerné ☐ 5.Refus
Menacé de faire mal à votre (dernière) partenaire ou à quelqu'un qui lui est proche ?	☐ 1.Oui → ☐ 2.Non	☐ 1.Souvent ☐ 2.Parfois ☐ 3.Jamais ☐ 4.Non concerné ☐ 5.Refus
Insulté ou rabaissé votre (dernière) partenaire ?	☐ 1.Oui → ☐ 2.Non	☐ 1.Souvent ☐ 2.Parfois ☐ 3.Jamais ☐ 4.Non concerné ☐ 5.Refus

Ne pas citer les mentions "Refus"

Q095 - Avez-vous déjà fait une des choses suivantes à votre (dernière) partenaire ?		Au cours des 12 derniers mois, cela est-il arrivé :
L'avez-vous déjà bousculée, secouée, ou avez-vous jeté quelque chose contre elle ?	☐ 1.Oui → ☐ 2.Non	☐ 1.Souvent ☐ 2.Parfois ☐ 3.Jamais ☐ 4.Non concerné ☐ 5.Refus
L'avez-vous déjà giflée ?	☐ 1.Oui → ☐ 2.Non	☐ 1.Souvent ☐ 2.Parfois ☐ 3.Jamais ☐ 4.Non concerné ☐ 5.Refus
Lui avez-vous déjà tordu le bras ou tiré les cheveux ?	☐ 1.Oui → ☐ 2.Non	☐ 1.Souvent ☐ 2.Parfois ☐ 3.Jamais ☐ 4.Non concerné ☐ 5.Refus
L'avez-vous déjà frappée à coup de poing ou avec quelque chose pour la blesser ?	☐ 1.Oui → ☐ 2.Non	☐ 1.Souvent ☐ 2.Parfois ☐ 3.Jamais ☐ 4.Non concerné ☐ 5.Refus
Lui avez-vous déjà donné des coups de pied, l'avez-vous déjà traînée à terre, ou l'avez-vous battue ?	☐ 1.Oui → ☐ 2.Non	☐ 1.Souvent ☐ 2.Parfois ☐ 3.Jamais ☐ 4.Non concerné ☐ 5.Refus
Avez-vous déjà essayé de l'étrangler ou de la brûler ?	☐ 1.Oui → ☐ 2.Non	☐ 1.Souvent ☐ 2.Parfois ☐ 3.Jamais ☐ 4.Non concerné ☐ 5.Refus
L'avez-vous déjà menacée avec un couteau, un pistolet ou un autre type d'arme ?	☐ 1.Oui → ☐ 2.Non	☐ 1.Souvent ☐ 2.Parfois ☐ 3.Jamais ☐ 4.Non concerné ☐ 5.Refus
L'avez-vous déjà forcée physiquement à avoir des rapports sexuels avec vous quand elle ne voulait pas ?	☐ 1.Oui → ☐ 2.Non	☐ 1.Souvent ☐ 2.Parfois ☐ 3.Jamais ☐ 4.Non concerné ☐ 5.Refus
L'avez-vous déjà forcée à pratiquer des actes sexuels qu'elle ne voulait pas ?	☐ 1.Oui → ☐ 2.Non	☐ 1.Souvent ☐ 2.Parfois ☐ 3.Jamais ☐ 4.Non concerné ☐ 5.Refus

Q096 - Est-ce qu'il vous est déjà arrivé que votre (dernière) partenaire vous frappe, gifle, donne des coups de pied ou vous fasse quelque chose d'autre pour vous faire mal physiquement alors que vous ne l'aviez ni battue, ni agressée physiquement ?

- ☐ 1.Oui
- ☐ 2.Non
- ☐ 3.Refus de répondre (ne pas citer)

→ **Passer directement au module H (page 32)**

A NE POSER QUE SI LE PATIENT EST UNE FEMME

Je vais maintenant vous poser des questions personnelles concernant la maternité, le désir d'enfant, les éventuelles interruptions de grossesses que vous pouvez avoir eues dans votre vie et enfin, les relations au sein du couple. Je vous rappelle que vos réponses sont confidentielles et anonymes et que je suis tenu(e) au secret professionnel.

Q088_F - Avez-vous des enfants ?

- ☐ 1.Oui → **Combien ?** |__|__|
  - ☐ 1.Ne sait pas (ne pas citer)
  - ☐ 2.Refus de répondre (ne pas citer)
- ☐ 2.Non
- ☐ 3.Refus de répondre (ne pas citer)

Q097 - Actuellement, souhaitez-vous ou essayez-vous d'avoir un enfant ?

- ☐ 1.Oui
- ☐ 2.Non
- ☐ 3.Non concernée (femme ménopausée etc.)
- ☐ 4.Ne sait pas (ne pas citer)
- ☐ 5.Refus de répondre (ne pas citer)

Q098 - Avez-vous déjà eu une grossesse qui n'a pas abouti ?

- ☐ 1.Oui → **Poser la question Q098_1**
- ☐ 2.Non
- ☐ 3.Refus de répondre (ne pas citer)

(2, 3) → **Passer à la question Q099**

Q098_1 - Est-ce que l'interruption de grossesse a nécessité — Une seule réponse possible

- ☐ 1.Un acte (chirurgical ou invasif) en milieu médical
- ☐ 2.Un acte (chirurgical ou invasif) en milieu non médical
- ☐ 3.Non, l'interruption de grossesse n'a pas nécessité d'acte chirurgical
- ☐ 4.Refus de répondre (ne pas citer)

Maintenant, si vous le permettez, je vais vous poser des questions concernant vos relations avec votre partenaire actuel(le) ou votre dernier(e) partenaire si vous n'avez pas de partenaire en ce moment.

Si la patiente répond spontanément qu'elle n'a jamais eu de partenaire, cocher la case "N'a jamais eu de partenaire" et passer directement au module H (page 32).

N'a jamais eu de partenaire ☐

Q099 - Qui décide habituellement comment l'argent que vous gagnez va être utilisé ?

Ne pas citer les modalités, laissez la personne répondre spontanément

- ☐ 1.L'enquêtée
- ☐ 2.Le mari/ partenaire
- ☐ 3.L'enquêtée et mari/ partenaire ensemble
- ☐ 4.Quelqu'un d'autre
- ☐ 5.L'enquêtée et quelqu'un d'autre ensemble
- ☐ 6.Autre **Précisez** ____________ Ecrire en majuscule
- ☐ 7.Refus de répondre (ne pas citer)

Q100 - Qui habituellement a le dernier mot dans les décisions concernant vos propres soins de santé ?

Ne pas citer les modalités, laissez la personne répondre spontanément

- ☐ 1.L'enquêtée
- ☐ 2.Le mari/ partenaire
- ☐ 3.L'enquêtée et mari/ partenaire ensemble
- ☐ 4.Quelqu'un d'autre
- ☐ 5.L'enquêtée et quelqu'un d'autre ensemble
- ☐ 6.Autre **Précisez** ____________ Ecrire en majuscule
- ☐ 7.Refus de répondre (ne pas citer)

Ne pas citer les mentions "Refus"

Q101 - Est-ce qu'il est déjà arrivé que votre (dernier) partenaire		Au cours des 12 derniers mois, cela est-il arrivé :		
Vous dise ou fasse quelque chose pour vous humilier devant d'autres personnes ?	☐ 1.Oui → ☐ 2.Non	☐ 1.Souvent ☐ 4.Non concernée	☐ 2.Parfois ☐ 5.Refus	☐ 3.Jamais
Vous menace, vous ou quelqu'un proche de vous ?	☐ 1.Oui → ☐ 2.Non	☐ 1.Souvent ☐ 4.Non concernée	☐ 2.Parfois ☐ 5.Refus	☐ 3.Jamais
Vous insulte ou vous rabaisse ?	☐ 1.Oui → ☐ 2.Non	☐ 1.Souvent ☐ 4.Non concernée	☐ 2.Parfois ☐ 5.Refus	☐ 3.Jamais

Ne pas citer les mentions "Refus"

Q102 - Est-ce qu'il est déjà arrivé que votre (dernier) partenaire vous fasse une des choses suivantes ?		Au cours des 12 derniers mois, cela est-il arrivé :		
Vous bouscule, secoue, ou jette quelque chose contre vous ?	☐ 1.Oui → ☐ 2.Non	☐ 1.Souvent ☐ 4.Non concernée	☐ 2.Parfois ☐ 5.Refus	☐ 3.Jamais
Vous gifle ?	☐ 1.Oui → ☐ 2.Non	☐ 1.Souvent ☐ 4.Non concernée	☐ 2.Parfois ☐ 5.Refus	☐ 3.Jamais
Vous torde le bras ou vous tire les cheveux ?	☐ 1.Oui → ☐ 2.Non	☐ 1.Souvent ☐ 4.Non concernée	☐ 2.Parfois ☐ 5.Refus	☐ 3.Jamais
Vous frappe à coups de poing ou avec quelque chose qui peut vous blesser ?	☐ 1.Oui → ☐ 2.Non	☐ 1.Souvent ☐ 4.Non concernée	☐ 2.Parfois ☐ 5.Refus	☐ 3.Jamais
Vous donne des coups de pieds, vous traîne à terre ou vous batte ?	☐ 1.Oui → ☐ 2.Non	☐ 1.Souvent ☐ 4.Non concernée	☐ 2.Parfois ☐ 5.Refus	☐ 3.Jamais
Essaye de vous étrangler ou de vous brûler ?	☐ 1.Oui → ☐ 2.Non	☐ 1.Souvent ☐ 4.Non concernée	☐ 2.Parfois ☐ 5.Refus	☐ 3.Jamais
Vous menace avec un couteau, un pistolet ou un autre type d'arme ?	☐ 1.Oui → ☐ 2.Non	☐ 1.Souvent ☐ 4.Non concernée	☐ 2.Parfois ☐ 5.Refus	☐ 3.Jamais
Vous force physiquement à avoir des rapports sexuels même quand vous ne le vouliez pas ?	☐ 1.Oui → ☐ 2.Non	☐ 1.Souvent ☐ 4.Non concernée	☐ 2.Parfois ☐ 5.Refus	☐ 3.Jamais
Vous force à pratiquer d'autres types d'actes sexuels que vous ne vouliez pas ?	☐ 1.Oui → ☐ 2.Non	☐ 1.Souvent ☐ 4.Non concernée	☐ 2.Parfois ☐ 5.Refus	☐ 3.Jamais

Q103 - Est-ce qu'il vous est déjà arrivé de battre, gifler, de donner des coups de pied ou de faire quelque chose d'autre pour agresser physiquement votre (dernier) partenaire alors qu'il ne vous avait ni battue, ni agressée physiquement ?

☐ 1.Oui

☐ 2.Non

☐ 3.Refus de répondre (ne pas citer)

MODULE H: SANTÉ PERÇUE ET QUALITÉ DE VIE

Je vais à présent vous poser des questions sur votre état de santé en général et sur l'impact que peut avoir la maladie dans votre vie de tous les jours.

Q104 - Dans l'ensemble, pensez-vous que votre santé est

- ☐ 1.Excellente
- ☐ 2.Très bonne
- ☐ 3.Bonne
- ☐ 4.Médiocre
- ☐ 5.Mauvaise
- ☐ 6.Refus de répondre (ne pas citer)

Q105 - Voici une liste d'activités que vous pouvez avoir à faire dans votre vie de tous les jours. Pour chacune d'entre elles indiquez si vous êtes gêné(e) en raison de votre état de santé actuel

Une réponse par ligne	1.Oui, beaucoup	2. Oui, un peu	3.Non, pas du tout	(ne pas citer) 4.Refus
Efforts physiques modérés tels que balayer le sol, déplacer une table, marcher 20 minutes sur un terrain plat	☐	☐	☐	☐
Monter plusieurs étages par l'escalier ou monter une pente très raide pendant quelques minutes	☐	☐	☐	☐

Q106 - Au cours de ces 4 dernières semaines, et en raison de votre état physique

Une réponse par ligne	1.En permanence	2.Très souvent	3. Souvent	4. Quelquefois	5. Rarement	6. Jamais	7. Refus
Avez-vous fait moins de choses que ce que vous auriez souhaité ?	☐	☐	☐	☐	☐	☐	☐
Avez-vous dû arrêter de faire certaines choses ?	☐	☐	☐	☐	☐	☐	☐

Q107 - Au cours de ces 4 dernières semaines, et en raison de votre état émotionnel (vous sentir triste, nerveux(se) ou déprimé(e))

Une réponse par ligne	1.En permanence	2.Très souvent	3. Souvent	4. Quelquefois	5. Rarement	6. Jamais	7. Refus
Avez-vous fait moins de choses que ce que vous auriez souhaité ?	☐	☐	☐	☐	☐	☐	☐
Avez-vous eu des difficultés à faire ce que vous aviez à faire avec autant de soin et d'attention ?	☐	☐	☐	☐	☐	☐	☐

Q108 - Au cours de ces 4 dernières semaines, dans quelle mesure vos douleurs physiques vous ont-elles gêné(e) dans votre travail ou vos activités quotidiennes ?

- ☐ 1.Pas du tout
- ☐ 2.Un petit peu
- ☐ 3.Moyennement
- ☐ 4.Beaucoup
- ☐ 5.Enormément
- ☐ 6.Refus de répondre (ne pas citer)

Q109 - Au cours de ces 4 dernières semaines, y a-t-il eu des moments où votre état de santé, physique ou émotionnel, vous a gêné(e) dans votre vie et vos relations avec les autres, votre famille, vos amis, vos connaissances ?

- ☐ 1.Tout le temps
- ☐ 2.Une grande partie du temps
- ☐ 3.De temps en temps
- ☐ 4.Rarement
- ☐ 5.Refus de répondre (ne pas citer)

Les questions qui suivent portent sur comment vous vous êtes senti(e) au cours de ces 4 dernières semaines. (Pour chaque question, veuillez indiquer la réponse qui vous semble la plus appropriée).

Q110 - Au cours de ces 4 dernières semaines, y a-t-il eu des moments où

Une réponse par ligne	1. En permanence	2.Très souvent	3. Souvent	4. Quelquefois	5. Rarement	6. Jamais	(ne pas citer) 7. Refus
Vous vous êtes senti calme et détendu ?	☐	☐	☐	☐	☐	☐	☐
Vous vous êtes senti débordant d'énergie ?	☐	☐	☐	☐	☐	☐	☐
Vous vous êtes senti triste et abattu ?	☐	☐	☐	☐	☐	☐	☐

Dans les questions suivantes, je vais vous demander de dire quel impact votre séropositivité a eu sur votre santé et votre vie. Il faut que vous pensiez à ce qu'a été votre vie au cours des deux dernières semaines.

Q111 - Au cours des deux dernières semaines, à cause de votre séropositivité

Une réponse par ligne	1. Jamais	2. Rarement	3.De temps en temps	4. Souvent	5.Tout le temps	6. Refus
Vous avez été gêné(e) par un changement de poids	☐	☐	☐	☐	☐	☐
Vous avez été gêné(e) par des problèmes de peau (peau sèche, démangeaisons, éruption)	☐	☐	☐	☐	☐	☐
Vous avez été gêné(e) par les changements d'aspect de votre corps (joues creuses, jambes, bras, fesses plus maigres, poitrine ou seins plus gros, gros ventre, bosse de graisse sur la nuque)	☐	☐	☐	☐	☐	☐
Vous avez été mécontent(e) de l'aspect de votre corps	☐	☐	☐	☐	☐	☐

MODULE I : LIENS SOCIAUX, DÉVOILEMENT DE LA SÉROPOSITIVITÉ, EXPÉRIENCE DES DISCRIMINATIONS

Nous allons maintenant parler plus particulièrement de vos expériences avec votre entourage à propos de votre infection VIH.

Q112 - Depuis que vous connaissez votre statut, l'avez-vous dit à quelqu'un ?

☐ 1.Oui
☐ 2.Non
☐ 3.Refus de répondre (ne pas citer)

→ **Passer directement à la question Q115 (page suivante)**

Je vais commencer par vous poser des questions sur la révélation de votre statut à votre entourage.

Q113 - Pour chacune des personnes que je vais vous citer, je vais vous demander si elles savent ou non que vous vivez avec le VIH. Pour les personnes qui le savent, je vais ensuite vous demander si c'est vous qui le leur avez dit ou pas et si cela est accepté ou pas.

NSP = Ne sait pas (ne pas citer)

Si Oui :

**Est-ce que (...........) sait que vous avez le VIH ?**	1.Non concerné	2.Oui	3.Non	4.NSP	**Vous le lui avez dit ?** 1.Oui	2.Non	**C'est accepté ?** 1.Oui	2.Non	3.NSP
Votre époux, conjoint, partenaire principal	☐	☐	☐	☐	☐	☐	☐	☐	☐
Votre père	☐	☐	☐	☐	☐	☐	☐	☐	☐
Votre mère	☐	☐	☐	☐	☐	☐	☐	☐	☐
Un frère, une sœur	☐	☐	☐	☐	☐	☐	☐	☐	☐
Votre/ vos enfants	☐	☐	☐	☐	☐	☐	☐	☐	☐
D'autres membres de votre famille	☐	☐	☐	☐	☐	☐	☐	☐	☐
Un ou plusieurs amis proches	☐	☐	☐	☐	☐	☐	☐	☐	☐
Un prêtre pasteur, imam ou autre religieux	☐	☐	☐	☐	☐	☐	☐	☐	☐
Un collègue de travail ou votre employeur	☐	☐	☐	☐	☐	☐	☐	☐	☐
Un de vos voisins	☐	☐	☐	☐	☐	☐	☐	☐	☐

Q114 - Avez-vous déjà demandé à quelqu'un de vous aider à annoncer votre statut sérologique ?

☐ 1.Oui → **A qui ?** ____________________ Ecrire en majuscule
☐ 2.Non
☐ 3.Refus de répondre (ne pas citer)

Dans les questions suivantes, nous vous demandons de dire quel impact votre séropositivité a eu sur votre vie sociale.

	1.Oui	2.Non	3.Refus de répondre **(ne pas citer)**
**Q115 - Avez-vous l'impression que certaines personnes ont peur parce que vous avez le VIH ?**	☐	☐	☐
**Q116 - Avez-vous perdu des amis après leur avoir dit que vous étiez séropositif(tive) ?**	☐	☐	☐
**Q117 - Y a-t-il des personnes qui sont proches de vous qui ont peur d'être rejetées parce que vous avez le VIH ?**	☐	☐	☐
**Q118 - Êtes-vous blessé(e) par la façon dont les gens réagissent quand ils apprennent que vous avez le VIH ?**	☐	☐	☐
**Q119 - Y a-t-il des personnes qui savent que vous avez le VIH et qui sont devenues plus distantes ?**	☐	☐	☐
**Q120 - Y a-t-il des gens auxquels vous tenez et qui ont arrêté de vous appeler quand ils ont appris que vous avez le VIH ?**	☐	☐	☐
**Q121 - Y a-t-il certaines personnes qui ont déjà eu une réaction de recul devant vous ?**	☐	☐	☐
**Q122 - Y a-t-il certaines personnes qui agissent comme si c'était votre faute si vous avez le VIH ?**	☐	☐	☐
**Q123 - Avez-vous vécu séparé de vos enfants et/ ou de votre famille depuis que vous avez appris que vous avez le VIH ?**	☐	☐	☐
**Q124 - Vous est-il arrivé de perdre votre emploi/ travail à cause de votre séropositivité ?**	☐	☐	☐

Q125 - Actuellement, diriez-vous que vous êtes moralement soutenu(e)

Une réponse par ligne	1. Pas du tout	2. Un peu	3. Moyennement	4. Beaucoup	5. Non concerné	**(ne pas citer)** 6. Refus
Par la personne avec qui vous vivez en couple (ou votre partenaire principal)	☐	☐	☐	☐	☐	☐
Par les membres de votre famille (parents et grands-parents, frères ou sœurs, cousins)	☐	☐	☐	☐	☐	☐
Par vos connaissances/ amis	☐	☐	☐	☐	☐	☐
Par votre communauté religieuse	☐	☐	☐	☐	☐	☐

Q126 - Est-ce que vous vous sentez seul(e) ?

☐ 1.Oui

☐ 2.Non

☐ 3.Ne sait pas (ne pas citer)

☐ 4.Refus de répondre (ne pas citer)

Q127 - Avez-vous des connaissances/ amis vivant avec le VIH que vous fréquentez/ avec lesquels vous sortez ?

☐ 1.Oui → **Poser la question Q127_1**

☐ 2.Non

☐ 3.Refus de répondre (ne pas citer)

→ **Passer à la question Q128**

Q127_1 - Combien ?

☐ 1.Une seule

☐ 2.De 2 à 5

☐ 3.De 6 à 10

☐ 4.Plus de 10

☐ 5.Ne sait pas (ne pas citer)

☐ 6.Refus de répondre (ne pas citer)

Q128 - Au cours des 12 derniers mois, avez-vous participé aux activités d'associations de lutte contre le sida (groupe de paroles, soirées, ateliers…) ?

☐ 1.Oui

☐ 2.Non

☐ 3.Ne sait pas (ne pas citer)

☐ 4.Refus de répondre (ne pas citer)

Noter l'heure de fin de l'entretien

E8 - Heure de fin de l'entretien *(ex: 11:26)* |h|h| : |m|m|

Analyse des évolutions du programme camerounais d'accès aux ARV dans les régions du Centre et du Littoral face aux enjeux actuels de la prise en charge du VIH et des principales co-infections (EVOLCAM)

- 2014 -
