# Supplementary material for: Intimate partner violence by men living with HIV in Cameroon: Prevalence, associated factors and implications for HIV transmission risk (ANRS-12288 EVOLCAM)
Source: PLoS One. 2021 Feb 18;16(2):e0246192. doi: 10.1371/journal.pone.0246192 (PMC7891744; doi:10.1371/journal.pone.0246192)
Supplement: S2 File — (PDF) [file pone.0246192.s002.pdf]

VERSION ANGLOPHONE

MODULE A : CARACTÉRISTIQUES SOCIODÉMOGRAPHIQUES ET ÉCONOMIQUES DU MÉNAGE

Q001 - Date of birth *(jj/mm/aaaa)* ____________

Q002 - What is the highest class you successfully attended ?

On entend par classe suivie avec succès, une année scolaire sanctionnée par le passage dans la classe supérieure et/ou l'acquisition d'un diplôme. Cocher une seule case

Système francophone		Système anglophone	
☐ 01.	Never fréquenté l'école	01.	Never gone to school
☐ 02.	Maternelle/ SIL	02.	Nursery/ Class1/ Class 2
☐ 03.	CP/ CPS	03.	Class 3
☐ 04.	CE1	04.	Class 4
☐ 05.	CE2	05.	Class 5
☐ 06.	CM1	06.	Class 6
☐ 07.	CM2	07.	Class 7
☐ 08.	6ème G ou 1ère A.T	08.	Form 1
☐ 09.	5ème G ou 2ème A.T	09.	Form 2
☐ 10.	4ème G ou 3ème A.T	10.	Form 3
☐ 11.	3ème G ou 4ème A.T ou BEPC ou CAP	11.	Form 4
☐ 12.	2nde G ou T	12.	Form 5
☐ 13.	1ère G ou T	13.	Lower sixth
☐ 14.	Terminale G ou T ou Bac	14.	Upper sixth
☐ 15.	1ère Year Univ./ IUT/ BTS	15.	1st year Univ.
☐ 16.	2ème Year Univ./ IUT/ BTS	16.	2nd year Univ.
☐ 17.	3ème Year Univ.	17.	3rd year Univ.
☐ 18.	4ème Year Univ.	18.	4th year Univ.
☐ 19.	5ème Year Univ. ou plus	19.	5th year Univ. or more
☐ 20.	Other →		
☐ 21.	Refus de répondre (ne pas citer)		

Specify ____________________

Ecrire en majuscule

VERSION ANGLOPHONE

Q003 - Where do you live? Ne pas citer. Pour Douala ou Yaoundé préciser le quartier. Ecrire en majuscule

☐ 1.In the town where the hospital is situated	☐ 2.Outside the town where the hospital is situated	☐ 3.Refus de répondre (ne pas citer)
**Q003_3 - If Douala or Yaoundé, specify the neighborhood**<br>______<br>☐ 1.Ne sait pas (ne pas citer)<br>☐ 2.Refus de répondre (ne pas citer)	**Q003_1 - Specify the Division/Dpt**<br>______<br>☐ 1.Ne sait pas (ne pas citer)<br>☐ 2.Refus de répondre (ne pas citer)<br>**Q003_2 - Specify the name of the town or village**<br>______<br>☐ 1.Ne sait pas (ne pas citer)<br>☐ 2.Refus de répondre (ne pas citer)<br>**Q003_3 - If Douala or Yaoundé, specify the neighborhood**<br>______<br>☐ 1.Ne sait pas (ne pas citer)<br>☐ 2.Refus de répondre (ne pas citer)	→ **Passer à la question Q004**

CONSIGNE DE SAISIE :

Ne pas poser au patient. A remplir au moment de la saisie du questionnaire

Q003_REC - Le patient habite en

- ☐ 1.Milieu urbain
- ☐ 2.Milieu semi-urbain
- ☐ 3.Milieu rural

Q004 - How many adults and children (including yourself) live within your household?

Q004_1 - Number of adults (18 years and above) ______

- ☐ 1.Ne sait pas (ne pas citer)
- ☐ 2.Refus de répondre (ne pas citer)

Q004_2 - Number of children (under 18 years) ______

- ☐ 1.Ne sait pas (ne pas citer)
- ☐ 2.Refus de répondre (ne pas citer)

VERSION ANGLOPHONE

Q005 - How are you related to the head of household ? Une seule réponse possible

- ☐ 1.You are the head of household → **Aller à Q006 (page suivante)**
- ☐ 2.You are the wife (or the husband)
- ☐ 3.You are his/her brother
- ☐ 4.You have an ascending family tie (father, mother, grand-father, grand-mother, uncle, aunt)
- ☐ 5.You have a descending family tie (son, daughter, grandson, grand-daughter, nephew, niece)
- ☐ 6.Other family ties (cousins)
- ☐ 7.No family tie **Specify** ____________________ Ecrire en majuscule
- ☐ 8.Refus de répondre (ne pas citer)

Si la personne interrogée n'est PAS le chef de ménage (Q005 > 1), répondre à l'encadré suivant :

Q005_1 - What is the current professional situation of the family/household head? Une seule réponse possible

- ☐ 1.Active, Occupied (he/she is gainfully employed/has an income) → **Répondre à l'encadré 1**
- ☐ 2.Retired/ Pensioner/Elderly Person
- ☐ 3.Joblessness, job seeker
- ☐ 4.Waiting to resume work once his/her health improves

(2 à 4) → **Répondre à l'encadré 2**

- ☐ 5.Other (Sans activité économique, Female/Male au foyer, étudiant) **Specify** ____________ Ecrire en majuscule
- ☐ 6.Ne sait pas (ne pas citer)
- ☐ 7.Refus de répondre (ne pas citer)

(5 à 7) → **Aller à Q006 (page suivante)**

Encadré 1 :
Si le chef de ménage est actif (Q005_1 = 1)

Q005_2 - What is the principal economic activity of the family/household head?

Demander au patient de décrire ce que fait le chef de ménage comme activité. Ecrire en majuscule

____

____

____

____

____

____

____

____

- ☐ 1.Ne sait pas (ne pas citer)
- ☐ 2.Refus de répondre (ne pas citer)

Q005_3 - Is it a regular (permanent) or casual (temporary) employment?

- ☐ 1.Regular
- ☐ 2.Casual
- ☐ 3.Ne sait pas (ne pas citer)
- ☐ 4.Refus de répondre (ne pas citer)

Encadré 2 :
Si le chef de ménage est inactif (Q005_1 = 2, 3 ou 4)

Q005_4 - What was the last economic activity carried out by the family/household head?

Demander au patient de décrire ce que faisait le chef de ménage comme activité quand il travaillait. Ecrire en majuscule

____

____

____

____

____

____

____

____

- ☐ 1.Ne sait pas (ne pas citer)
- ☐ 2.Refus de répondre (ne pas citer)

Q005_5 - Was it a regular or an casual economic activity?

- ☐ 1.Regular
- ☐ 2.Casual
- ☐ 3.Ne sait pas (ne pas citer)
- ☐ 4.Refus de répondre (ne pas citer)

VERSION ANGLOPHONE

Q006 - How many persons amongst your household members (including yourself) contribute to the household income?

_______ persons ☐ 1.Ne sait pas (ne pas citer) ☐ 2.Refus de répondre (ne pas citer)

Q007 - What was your household income in the previous month, (taking into account the financial contribution of every family member and all sources of income- such as aid from the family, pensions, land sales, etc.) ?

_______________ FCFA/month

Si le patient répond "ça dépend des mois" et donne plusieurs montants, cocher "Ne sait pas" et énumérer l'échelle Q007_1

En cas de réponse spontanée, aller à Q008 sans énumérer l'échelle

☐ 1.Ne sait pas (ne pas citer) ☐ 2.Refus de répondre (ne pas citer)

Q007_1 - Could you indicate/estimate the approximate monthly income of your household in the previous month?

Une seule réponse possible

- ☐ 01.Less than 10 000 CFA
- ☐ 02.Between 10 000 and 30 000 CFA
- ☐ 03.Between 30 000 and 50 000 CFA
- ☐ 04.Between 50 000 and 70 000 CFA
- ☐ 05.Between 70 000 and 100 000 CFA
- ☐ 06.Between 100 000 and 150 000 CFA
- ☐ 07.Between 150 000 and 300 000 CFA
- ☐ 08.Between 300 000 and 500 000 CFA
- ☐ 09.Over 500 000 CFA
- ☐ 10.Ne sait pas (ne pas citer)
- ☐ 11.Refus de répondre (ne pas citer)

Q008 - During the previous month, approximately how much has your household spent on

Inscrire 0 si aucune dépense

		(ne pas citer) 1.Ne sait pas	(ne pas citer) 2.Refus de répondre
Food (incl. water/ maintenance/ hygiene)	_______ FCFA	☐	☐
Housing (rents+ electricity + little maintenance jobs)	_______ FCFA	☐	☐
Recreational activities, outings and leisure (bars etc.)/ trips	_______ FCFA	☐	☐
Transportation (taxis, fuel, etc.)	_______ FCFA	☐	☐
Communication (phone bills)	_______ FCFA	☐	☐

Q009 - During the last 6 months, approximately how much has your household spent on

Inscrire 0 si aucune dépense

		(ne pas citer) 1.Ne sait pas	(ne pas citer) 2.Refus de répondre
Education (school fees, books, ...)	_______ FCFA	☐	☐
Clothing/ Haircare/ beauty treatments	_______ FCFA	☐	☐
Household goods (electrical appliances, furniture, ...)	_______ FCFA	☐	☐
Aiding family/ friends	_______ FCFA	☐	☐

VERSION ANGLOPHONE

Q010 - Is your household?

- ☐ 1.House owner
- ☐ 2.Tenant (you pay rents)
- ☐ 3.Other **Specify** ______________ Ecrire en majuscule
- ☐ 4.Ne sait pas (ne pas citer)
- ☐ 5.Refus de répondre (ne pas citer)

Q011 - Does your home have

Une réponse par ligne

	1.Yes	2.No	(ne pas citer) 3.Refus de répondre
Bathroom (that is running water with a shower)	☐	☐	☐
Flushable Toilet/WC	☐	☐	☐
Traditional/Pit Latrines	☐	☐	☐
A kitchen sink	☐	☐	☐
Electricity	☐	☐	☐
Air conditioning	☐	☐	☐
A Refrigerator	☐	☐	☐
A gas or Electric cooker with an oven	☐	☐	☐
Internet connection	☐	☐	☐

Q012 - What is the main material used in constructing the walls of your house?

- ☐ 1.Ciment/bricks
- ☐ 2.Boards/Carabot
- ☐ 3.Metalsheets/zinc
- ☐ 4.Mud bricks
- ☐ 5.Other **Specify** ______________ Ecrire en majuscule
- ☐ 6.Ne sait pas (ne pas citer)
- ☐ 7.Refus de répondre (ne pas citer)

Q013 - Does your household own the following?

Une réponse par ligne

	1.Yes	2.No	(ne pas citer) 3.Ne sait pas	4.Refus de répondre
Car, van	☐	☐	☐	☐
Television	☐	☐	☐	☐
Radio	☐	☐	☐	☐
Computer	☐	☐	☐	☐
One or more farmlands	☐	☐	☐	☐

Q014 - Over the past four weeks, have you had at least two meals a day Une seule réponse possible

- ☐ 1.Always
- ☐ 2.Almost always (an average of 5 to 6 days on 7)
- ☐ 3.Sometimes (averagely 4 to 5 days on 7)
- ☐ 4.Rarely (less than twice a week)
- ☐ 5.Never
- ☐ 6.Ne sait pas (ne pas citer)
- ☐ 7.Refus de répondre (ne pas citer)

MODULE B : ACTIVITÉ PROFESSIONNELLE DU PATIENT

I will now ask you questions about your professional activity.

Q015 - Do you currently work or do you practice an income generating activity, even if you earn very little ?

On considère comme travail toutes les activités qui permettent de gagner de l'argent, comme le travail aux champs (même s'il n'est pas payé, il permet d'avoir un revenu grâce à la vente des produits). Les activités ménagères à la maison ne sont pas considérées comme un travail

- ☐ 1.Yes
- ☐ 2.No, not at all
- ☐ 3.Refus de répondre (ne pas citer)

Si Oui (Q015 = 1), répondre à l'encadré ci-dessous :

Q015_1 - What is your current main economic activity?

Demander au patient de décrire ce qu'il fait. Ecrire en majuscule

____

- ☐ 1.Ne sait pas (ne pas citer)
- ☐ 2.Refus (ne pas citer)

Q015_2 - Is it a regular or casual employment?

- ☐ 1.Regular
- ☐ 2.Casual
- ☐ 3.Refus de répondre (ne pas citer)

Q015_3 - How many people work with you?

- ☐ 1.Between 1 and 10
- ☐ 2.More than 10
- ☐ 3.Refus de répondre (ne pas citer)

Q015_4 - You work as **Une seule réponse possible**

- ☐ 1.State employee
- ☐ 2.Salaried worker (whatever the sector, including salaried by a private individual)
- ☐ 3.Independent (with no dependant employees)
- ☐ 4.Head of enterprise (with at least one dependant employee)
- ☐ 5.Apprentice or unpaid family help
- ☐ 6.Other **Specify** _______________ **Ecrire en majuscule**
- ☐ 7.Refus de répondre (ne pas citer)

<u>During the past month</u>

Q015_5 - How much did you earn?

_______________ FCFA

- ☐ 1.Ne sait pas (ne pas citer)
- ☐ 2.Refus de répondre (ne pas citer)

Q015_6 - About how many days did you work?

_______ days

- ☐ 1.Ne sait pas (ne pas citer)
- ☐ 2.Refus de répondre (ne pas citer)

Q015_7 - When you worked, about how many hours a day did you work?

_______ hours/day

- ☐ 1.Ne sait pas (ne pas citer)
- ☐ 2.Refus de répondre (ne pas citer)

Q015_8 - Did you ever miss out on work because of ill-health?

- ☐ 1.Yes → **How many days?** _______ days
  - ☐ Ne sait pas combien (ne pas citer)
- ☐ 2.No
- ☐ 3.Refus de répondre (ne pas citer)

Si Non ou Refus (Q015 = 2 ou 3), répondre à l'encadré ci-dessous :

Q015_9 - What is your current situation? **Une seule réponse possible**

- ☐ 1.Student
- ☐ 2.House wife or pregnant woman/post pregnancy
- ☐ 3.Retired/ Pensioner/Elderly Person
- ☐ 4.Joblessness or in search of a job
- ☐ 5.Waiting to resume work when your health improves
- ☐ 6.Disabled/ permanent handicap
- ☐ 7.Other **Specify** _______________ **Ecrire en majuscule**
- ☐ 8.Refus de répondre (ne pas citer)

VERSION ANGLOPHONE

Q016 - During the past month did you receive any help in carrying out your daily tasks/activities (cooking, care of the children, taking care of yourself, shopping, etc) because you were sick ?

☐ 1.Yes ☐ 2.No ☐ 3.Refus de répondre (ne pas citer)

Passer à la question Q017 (page suivante)

Si Oui (Q016 = 1) répondre à l'encadré ci-dessous :

Q016_1 - Concerning the person who helped you during the past month (main help if several)

Q016_1_1 - Does this person who helped you live in your household ?

☐ 1.Yes
☐ 2.No
☐ 3.Refus de répondre (ne pas citer)

Q016_1_2 - About how old is the person who helped you?

_________ years

☐ 1.Ne sait pas (ne pas citer)
☐ 2.Refus de répondre (ne pas citer)

Q016_1_3 - Were you paying this person who helped you for the help provided?

☐ 1.Yes
☐ 2.No
☐ 3.Refus de répondre (ne pas citer)

Q016_2 - During the past month for how many days have you needed this person's help?

_______ days ☐ 1.Ne sait pas (ne pas citer) ☐ 2.Refus de répondre (ne pas citer)

Q016_3 - Was the person helping you in the past month required to stop carrying out their usual activity (either completely or partially)?

☐ 1.Yes
☐ 2.No
☐ 3.Not concerned (This person does not work)
☐ 4.Ne sait pas (ne pas citer)
☐ 5.Refus de répondre (ne pas citer)

MODULE C : HISTOIRE ET TRAITEMENT DE L'INFECTION À VIH

We will now talk about health problems, care and treatment you received starting from the moment you found out your seropositive status.

Q017 - When did you learn that the results of your HIV test was positive?

Demander la date précise ; inscrire la durée approximative si le patient n'a pas souvenir de la date exacte

Month _____ **Year** _____ **OR** **Number of months** _____ **Number of years** _____

☐ 1.Ne sait pas (ne pas citer) ☐ 2.Refus de répondre (ne pas citer)

Q018 - Did you do the test Une seule réponse possible

☐ 1.On your own initiative
☐ 2.At the request of a health provider
☐ 3.At the request of your partner
☐ 4.The test was done without your knowledge
☐ 5.Refus de répondre (ne pas citer)

Q019 - Under what circumstances did you do the test? Une seule réponse possible

☐ 1.Following the symptoms of an illness (fever, weight loss,...)
☐ 2.During pregnancy check ups (ANC) or during delivery
☐ 3.During an HIV testing campaign
☐ 4.Following the discovery of the infection of one of your relatives **Specify who** _____
☐ 5.Other **Specify** _____ Ecrire en majuscule
☐ 6.Refus de répondre (ne pas citer)

Q020 - After learning of your positive HIV status, how long did it take for you to see a health care provider as the follow up of your HIV infection?

☐ 1.Less than a month
☐ 2.Between 1 to 3 months
☐ 3.Between 3 and 6 months
☐ 4.Between 6 months and a year
☐ 5.More than a year
☐ 6.Refus de répondre (ne pas citer)

We are now going to talk on HIV treatment also called Antiretroviral treatment or ART.

Q021 - Do you think the ART treatment

NSP = Ne sait pas **(ne pas citer)**

Une réponse par ligne	1.Never	2.A bit	3.Enough	4.A lot	5.NSP	6.Refus
Reduces The Risk Of Transmitting HIV during sexual intercourse	☐	☐	☐	☐	☐	☐
Reduces the risk of transmitting HIV to an unborn baby during pregnancy	☐	☐	☐	☐	☐	☐

VERSION ANGLOPHONE

TRAITEMENTS ET SUIVI MÉDICAL

Q022 - On a scale of 1 to 6, do you generally respect all medical prescriptions given to you (all treatments combined)

1 (never) **Entourer le chiffre indiqué par le patient** (always) 6

1 2 3 4 5 6

☐ 1.Ne sait pas (ne pas citer) ☐ 2.Refus de répondre (ne pas citer)

Q023 - During the past month have you taken an ART treatment? ☐ Refus de répondre (ne pas citer)

☐ 1.Yes	☐ 2.No, but you have already received a treatment in the past	☐ 3.No, You have never received any treatment
**Q023_1 - When did you begin taking this treatment?**<br>Month ______ Year ______<br>☐ 1.Ne sait pas (ne pas citer)<br>☐ 2.Refus de répondre (ne pas citer)<br><br>**Q023_2 - During the course of the past 12 months, have you personally discussed this treatment with a counselor?**<br>**Le conseiller est différent du médecin. Il peut être infirmier, assistant social, psychologue**<br>☐ 1.Yes, once<br>☐ 2.Yes, several times<br>☐ 3.No<br>☐ 4.Refus de répondre (ne pas citer)<br><br>→ **Passer à la section OBSERVANCE page suivante (Q024)**	**Q023_3 - For how long had you been taking this treatment before stopping?**<br>Number of months ______<br>Number of years ______<br>☐ 1.Ne sait pas (ne pas citer)<br>☐ 2.Refus de répondre (ne pas citer)<br><br>**Q023_4 - For how long now have you stopped?**<br>☐ 1.Less than 3 months<br>☐ 2.Between 3 and 6 months<br>☐ 3.More than 6 months<br>☐ 4.Refus de répondre (ne pas citer)<br><br>→ **Passer au module D page 14**	**Q023_5 - Are you currently waiting for your medical exam result or an appointment with the doctor to initiate treatment soon?**<br>☐ 1.Yes<br>☐ 2.No<br>☐ 3.Refus de répondre (ne pas citer)<br><br>→ **Passer au module D page 14**

VERSION ANGLOPHONE

OBSERVANCE

Q024 - We are now interested in the ARV treatment you have taken over the past four days, starting from yesterday.

Utiliser les images de médicaments en couleur pour identifier les médicaments avec le patient

	What is/are the name(s) of your ART drug(s) for HIV? Ecrire en majuscule	How many tablets are prescribed a day by the doctor?	How many tablets did you take			
			Yesterday ?	2 days ago?	3 days ago?	4 days ago?
	Exemple : TRIOMUNE 30	*Exemple : 2 comprimés / jour*	*Exemple : 2*	*Exemple : 0*	*Exemple : 1*	*Exemple : 0*
**Drug No 1**						
**Drug No 2**						
**Drug No 3**						
**Drug No 4**						
**Drug No 5**						

Q025 - Over the past four days, has it ever happened that you have taken the daily does of your ART drugs all at once?

☐ 1.Yes, always as my treatment is carried out once a day
☐ 2.Yes, several times
☐ 3.Yes, once
☐ 4.No, never
☐ 5.Refus de répondre (ne pas citer)

Q026 - Over the past four days, has it ever happened that you forgot or delayed taking your ART drugs for several hours after the required time?

☐ 1.Yes, several times
☐ 2.Yes, once
☐ 3.No, never
☐ 4.Refus de répondre (ne pas citer)

Q027 - Over the past four days, concerning your ART treatment, have you Une seule réponse possible

☐ 1.Stopped all treatment at the request of the doctor
☐ 2.Stopped all treatment for personal reasons
☐ 3.Treatment discontinued due to ARV stock-out
☐ 4.Continued to partially follow treatment
☐ 5.Continued to fully follow treatment
☐ 6.Refus de répondre (ne pas citer)

Q028 - It has been noticed that it is often more difficult for people to take their ART drugs during the weekend (Saturday-Sunday). Did you miss one or more tablets of your ART treatment for HIV this past week-end?

☐ 1.Yes
☐ 2.No
☐ 3.Refus de répondre (ne pas citer)

VERSION ANGLOPHONE

We will now be interested in the ART treatment for HIV you have taken over the past four weeks.

Q029 - In your opinion, over the past four weeks, you have **Une seule réponse possible**

- ☐ 1.Scrupuliously respected medical prescriptions
- ☐ 2.Globally respected medical prescriptions with some deviation
- ☐ 3.Sometimes modified the intakes (quantities and periods)
- ☐ 4.Practically never respected medical prescriptons
- ☐ 5.Stopped all treatement
- ☐ 6.Treatment stopped due to ARV stock-out
- ☐ 7.Refus de répondre (ne pas citer)

Q030 - Over the past four weeks, have ever you interrupted your treatment for more than two days ?

- ☐ 1.Yes, several times
- ☐ 2.Yes, once
- ☐ 3.No, never
- ☐ 4.Refus de répondre (ne pas citer)

Q031 - Over the past three months have you at any time not been able to find one or more of your ART drugs at the hospital pharmacy where you normally go to ?

- ☐ 1.Yes, at least once → **Poser la question Q031_1**
- ☐ 2.No, never → **Passer à la question Q032**
- ☐ 3.Refus de répondre (ne pas citer) → **Passer à la question Q032**

Q031_1 - Has this been within the past four weeks ?

- ☐ 1.Yes
- ☐ 2.No
- ☐ 3.Refus de répondre (ne pas citer)

Q032 - Over the past three months have you at any point in time had to pay to receive your ART treatment?

- ☐ 1.Yes, several times
- ☐ 2.Yes, once
- ☐ 3.No, never
- ☐ 4.Ne sait pas (ne pas citer)
- ☐ 5.Refus de répondre (ne pas citer)

Si Oui, plusieurs fois ou une fois (Q032 = 1 ou 2) répondre à l'encadré ci-dessous :

Q032_1 - Where did you buy these ARTs?

Une réponse par ligne

	1.Yes	2.No	3.Ne sait pas	4.Refus de répondre
In this hospital	☐	☐	☐	☐
In another hospital, dispensary or health center	☐	☐	☐	☐
In a pharmacy	☐	☐	☐	☐
By the roadside or in the market	☐	☐	☐	☐
Other **Specify** ____________	☐	☐	☐	☐

Ecrire en majuscule

VERSION ANGLOPHONE

Q033 - Over the past three months, have there been instances when you couldn't get your ART treatment due to lack of money?

☐ 1.Yes, several times

☐ 2.Yes, once

☐ 3.No, never

☐ 4.Refus de répondre (ne pas citer)

Q034 - Since you began taking an ART treatment, have you ever stopped this treatment for longer than a month?

☐ 1.Yes, at least once → **Poser les questions Q034_1 et Q034_2**

☐ 2.No, never

☐ 3.Refus de répondre (ne pas citer) → **Passer au module D (page suivante)**

Q034_1 - For how long did you stop your treatment?

☐ 1.Between 1 and 3 months

☐ 2.Between 3 months and one year

☐ 3.More than a year

☐ 4.Ne sait pas (ne pas citer)

☐ 5.Refus de répondre (ne pas citer)

Q034_2 - Was this in the past 6 months?

☐ 1.Yes

☐ 2.No

☐ 3.Ne sait pas (ne pas citer)

☐ 4.Refus de répondre (ne pas citer)

MODULE G : COMPORTEMENTS À RISQUE ET COMPORTEMENTS SEXUELS

We will now address more personal questions. We will begin with questions pertaining to your consumption of alcohol and other hard substances, be it occasionally or regularly, then move on to questions on sexuality.

CONSOMMATION D'ALCOOL ET AUTRES PRODUITS

Q072 - Do you drink beer?

☐ 1.Never → **Passer à la question Q073**
☐ 2.At least once a month
☐ 3.Between 2 to 4 times a month
☐ 4.Betweeen 2 to 3 times a week
☐ 5.Between 4 and 6 times a week
☐ 6.Everyday
☐ 7.Refus de répondre (ne pas citer) → **Passer à la question Q073**

Q072_1 - When drinking beer, about how many big bottles (65 centiliters) or small bottles (33cl) do you drink each time ?

☐ 1.At least one big bottle of 65cl (= less than 2 small bottles)
☐ 2.Between 2 to 3 big bottles of 65cl (= between 3 to 6 small bottles)
☐ 3.Between 4 to 5 big bottles of 65cl (= between 7 to 10 small bottles)
☐ 4.More than 5 big bottles of 65cl (= more than 11 small bottles)
☐ 5.Ne sait pas (ne pas citer)
☐ 6.Refus de répondre (ne pas citer)

Q073 - Have you ever drank more than 3 big bottles of beer and/or more than 6 glasses (or "sachets") of other alcoholic beverages at the same time ?

☐ 1.Never
☐ 2.Less than once a month
☐ 3.Once a month
☐ 4.Once per week
☐ 5.Almost everyday
☐ 6.Non concerné (ne pas citer)
☐ 7.Ne sait pas (ne pas citer)
☐ 8.Refus de répondre (ne pas citer)

Q074 - Have you ever consumed banga/ ndjap/ cannabis/ tramol/ caillou/ cocaine, even if only once in your life no matter how long ago?

☐ 1.Yes, in the past four weeks
☐ 2.Yes, at least once in your life
☐ 3.No
☐ 4.Refus de répondre (ne pas citer)

VERSION ANGLOPHONE

SEXUALITÉ

We will now move on to questions concerning your sexuality.

Q075 - Through the course of your life, how many different partners have you had sexual relations with (including your current partner)?

Demander le nombre de partenaires sexuels hommes ET le nombre de partenaires sexuels femmes au patient

Q075_1 - Number of men

- ☐ 1.None
- ☐ 2.One
- ☐ 3.Between 2 and 5
- ☐ 4.Between 6 and 10
- ☐ 5.Between 11 and 20
- ☐ 6.Between 21 and 50
- ☐ 7.More than 50
- ☐ 8.Ne sait pas (ne pas citer)
- ☐ 9.Refus de répondre (ne pas citer)

Q075_2 - Number of women

- ☐ 1.None
- ☐ 2.One
- ☐ 3.Between 2 and 5
- ☐ 4.Between 6 and 10
- ☐ 5.Between 11 and 20
- ☐ 6.Between 21 and 50
- ☐ 7.More than 50
- ☐ 8.Ne sait pas (ne pas citer)
- ☐ 9.Refus de répondre (ne pas citer)

Q076 - How old were you when you had what you consider your first sexual relation ?

_____ years ☐ 1.Ne sait pas (ne pas citer) ☐ 2.Refus de répondre (ne pas citer)

Q077 - Did you use a condom?

- ☐ 1.Yes
- ☐ 2.No
- ☐ 3.Ne sait pas (ne pas citer)
- ☐ 4.Refus de répondre (ne pas citer)

VERSION ANGLOPHONE

Q078 - Have you currently a spouse/ a boy/girlfriend/ a main partner? ☐ Refus de répondre (ne pas citer)

☐ 1.Yes	☐ 2.No
**Q078_1 - Are you**<br>☐ 1.Legally or 'customarily/traditionally' married<br>☐ 2.Cohabiting<br>☐ 3.Refus de répondre (ne pas citer)<br><br>**Q078_2 - Are you in a polygamous marriage/union?**<br>☐ 1.Yes<br>☐ 2.No<br>☐ 3.Refus de répondre (ne pas citer)<br><br>**Q078_3 - Do you live with your main partner /spouse in the same home ?**<br>☐ 1.Yes<br>☐ 2.No<br>☐ 3.Refus de répondre (ne pas citer)<br><br>**Q078_4 - For how long have you been together ?**<br>_______ month **OR** _______ years<br>☐ 1.Ne sait pas (ne pas citer)<br>☐ 2.Refus de répondre (ne pas citer)<br><br>**Q078_5 - What is the level of education of your spouse/partner?**<br>☐ 1.Never attended school<br>☐ 2.Primary (CP to CM2/Class 3 to Class 7)<br>☐ 3.Secondary 1st cycle (6ème à 3ème/ Form1 to Form4)<br>☐ 4.Secondary 2nd cycle (2nde, 1ère, Terminale/ Form 5, Lower Sixth, UpperSixth)<br>☐ 5.Universities or higher studies after GCE Advance Level<br>☐ 6.Ne sait pas (ne pas citer)<br>☐ 7.Refus de répondre (ne pas citer)	**Q078_6 - Are you**<br>☐ 1.Widow/Widower → **Poser la question Q078_7**<br>☐ 2.Single → **Poser la question Q078_7**<br>☐ 3.Divorced/Separated → **Poser la question Q078_8**<br>☐ 4.Refus de répondre (ne pas citer) → **Passer à la question Q079**<br><br>**Q078_7 - Over the past 12 months have you broken up with a main partner ?**<br>☐ 1.Yes → **Poser la question Q078_8**<br>☐ 2.No → **Passer à la question Q079**<br>☐ 3.Refus de répondre (ne pas citer) → **Passer à la question Q079**<br><br>**Q078_8 - Was this break-up in relation to your HIV status?**<br>☐ 1.Yes<br>☐ 2.No<br>☐ 3.Ne sait pas (ne pas citer)<br>☐ 4.Refus de répondre (ne pas citer)

Q079 - Over the past 12 months, how many sexual partners have you had ?

_______ sexual partners

En cas de réponse spontanée, ne pas énumérer l'échelle

Si le patient ne donne pas de chiffre précis, énumérer l'échelle suivante

☐ 1.No partner
☐ 2.One partner
☐ 3.From 2 to 5 partners
☐ 4.From 6 to 10 partrners
☐ 5.More than 10 partners
☐ 6.Ne sait pas (ne pas citer)
☐ 7.Refus de répondre (ne pas citer)

Si le patient a eu au moins un partenaire (Q079 > 0) passer à la question Q080 (page suivante).

Si le patient n'a eu aucun partenaire sexuel au cours des 12 derniers mois (Q079 = 0) ou n'a pas répondu à la question Q079, passer au module sur les violences :
→ **Si le patient est un homme, aller page 28 à la question Q088_H**
→ **Si le patient est une femme, aller page 30 à la question Q088_F**

VERSION ANGLOPHONE

I will now ask you a few personal questions concerning your sexual partners during the 12 last months.

Poser les questions jusqu'à 2 partenaires, en commençant par le plus récent
Ne pas citer les mentions "Ne sait pas" et "Refus de répondre"

	Most recent sexual partner	Last sexual partner
**Q080 - Your sexual partner was**	☐ 1.Main Partner<br>☐ 2.Casual Partner<br>☐ 3.Refus de répondre	☐ 1.Main Partner<br>☐ 2.Casual Partner<br>☐ 3.Refus de répondre
**Q081 - How old is he/she?**	☐ 1.About the same age as you (plus or minus 5 years)<br>☐ 2.Younger than you (Age difference of more than 5 years)<br>☐ 3.Older than you (Age difference of more than 5 years)<br>☐ 4.Ne sait pas<br>☐ 5.Refus de répondre	☐ 1.About the same age as you (plus or minus 5 years)<br>☐ 2.Younger than you (Age difference of more than 5 years)<br>☐ 3.Older than you (Age difference of more than 5 years)<br>☐ 4.Ne sait pas<br>☐ 5.Refus de répondre
**Q082 - Did you know his/her HIV status ?**	☐ 1.Yes, Seropositive<br>☐ 2.Yes, Seronegative<br>☐ 3.Ne sait pas<br>☐ 4.Refus de répondre	☐ 1.Yes, Seropositive<br>☐ 2.Yes, Seronegative<br>☐ 3.Ne sait pas<br>☐ 4.Refus de répondre
**Q083 - Did you reveal your HIV status to this partner?**	☐ 1.Yes<br>☐ 2.No<br>☐ 3.Ne sait pas<br>☐ 4.Refus de répondre	☐ 1.Yes<br>☐ 2.No<br>☐ 3.Ne sait pas<br>☐ 4.Refus de répondre
**Q084 - Can you say approximately, how many times you have had sex with this partner ?**	☐ 1.Less than once a month<br>☐ 2.Once a month<br>☐ 3.More than once a month<br>☐ 4.Several times a week<br>☐ 5.Everyday<br>☐ 6.Ne sait pas<br>☐ 7.Refus de répondre	☐ 1.Less than once a month<br>☐ 2.Once a month<br>☐ 3.More than once a month<br>☐ 4.Several times a week<br>☐ 5.Everyday<br>☐ 6.Ne sait pas<br>☐ 7.Refus de répondre
**Q085 - During your sexual relations with this partner did you use condoms?**	☐ 1.Never<br>☐ 2.From time to time<br>☐ 3.Almost always<br>☐ 4.Always<br>☐ 5.Ne sait pas<br>☐ 6.Refus de répondre	☐ 1.Never<br>☐ 2.From time to time<br>☐ 3.Almost always<br>☐ 4.Always<br>☐ 5.Ne sait pas<br>☐ 6.Refus de répondre
**Q086 - During your last sexual relation with this partner did you use condoms?**	☐ 1.Yes<br>☐ 2.No<br>☐ 3.Ne sait pas<br>☐ 4.Refus de répondre	☐ 1.Yes<br>☐ 2.No<br>☐ 3.Ne sait pas<br>☐ 4.Refus de répondre

Q087 - Over the past 12 months have you had sexual relations in which you paid for sex or you were paid to have sex?

☐ 1.Paying
☐ 2.Being paid or in exchange for a gift
☐ 3.No
☐ 4.Ne sait pas (ne pas citer)
☐ 5.Refus de répondre (ne pas citer)

VERSION ANGLOPHONE

VIOLENCE CONJUGALE ET SEXUELLE

A NE POSER QUE SI LE PATIENT EST UN HOMME

Si le patient est une femme passer directement à la question Q088_F (page 30)

I will now ask you a few personal questions concerning fatherhood, the desire for a child, and finally relations between couples. I want to remind you that all your responses are confidential and anonymous, and that I am bound by professional secrecy.

Q088_H - Do you have children?

- ☐ 1.Yes → **How many** ______ ☐ 1.Ne sait pas (ne pas citer) ☐ 2.Refus de répondre (ne pas citer)
- ☐ 2.No
- ☐ 3.Refus de répondre (ne pas citer)

Q089 - Do you currently wish or are you trying to have a child with your wife or partner ?

- ☐ 1.Yes
- ☐ 2.No
- ☐ 3.Not concerned (elderly man, etc.)
- ☐ 4.Ne sait pas (ne pas citer)
- ☐ 5.Refus de répondre (ne pas citer)

Q090 - Are you circumcised ?

- ☐ 1.Yes, in a traditional manner
- ☐ 2.Yes, medically
- ☐ 3.No
- ☐ 4.Refus de répondre (ne pas citer)

If you will permit me, I will now ask some questions concerning your relations with your current or last partner if you do not have a partner at the moment.

Si le patient répond spontanément qu'il n'a jamais eu de partenaire, cocher la case "N'a jamais eu de partenaire" et passer directement au module H (page 32).

N'a jamais eu de partenaire ☐

Q092 - Who usually makes decisions on how the money you earn is to be spent ?

Ne pas citer les modalités, laissez la personne répondre spontanément

- ☐ 1.Interviewee/respondent
- ☐ 2.Wife/Partner
- ☐ 3.Interviewee/respondent together with Wife/ Partner
- ☐ 4.Someone else
- ☐ 5.Interviewee/respondent together with somebody else
- ☐ 6.Other **Specify** ______ **Ecrire en majuscule**
- ☐ 7.Refus de répondre (ne pas citer)

Q093 - Who usually has the last word in decisions concerning your own health care?

Ne pas citer les modalités, laissez la personne répondre spontanément

- ☐ 1.Interviewee/respondent
- ☐ 2.Wife/Partner
- ☐ 3.Interviewee/respondent together with Wife/ Partner
- ☐ 4.Someone else
- ☐ 5.Interviewee/respondent together with somebody else
- ☐ 6.Other **Specify** ______ **Ecrire en majuscule**
- ☐ 7.Refus de répondre (ne pas citer)

VERSION ANGLOPHONE

Ne pas citer les mentions "Refus"

Q094 - Have you ever		During the past 12 months this has happened :
Said or done something to humiliate your (last) partner infront of other people?	☐ 1.Yes → ☐ 2.No	☐ 1.Often ☐ 2.Sometimes ☐ 3.Never ☐ 4.Not concerned ☐ 5.Refus
Threatened to harm your (last) partner or some body close to him/her?	☐ 1.Yes → ☐ 2.No	☐ 1.Often ☐ 2.Sometimes ☐ 3.Never ☐ 4.Not concerned ☐ 5.Refus
Insulted or belittled your (last) partner?	☐ 1.Yes → ☐ 2.No	☐ 1.Often ☐ 2.Sometimes ☐ 3.Never ☐ 4.Not concerned ☐ 5.Refus

Ne pas citer les mentions "Refus"

Q095 - Have you ever done any of the following to your (last) partner?		During the past 12 months this has happened :
Have you ever hit, shaken forcefully or thrown something at her ?	☐ 1.Yes → ☐ 2.No	☐ 1.Often ☐ 2.Sometimes ☐ 3.Never ☐ 4.Not concerned ☐ 5.Refus
Have you ever slapped her ?	☐ 1.Yes → ☐ 2.No	☐ 1.Often ☐ 2.Sometimes ☐ 3.Never ☐ 4.Not concerned ☐ 5.Refus
Have you ever twisted her arm or pulled her hair?	☐ 1.Yes → ☐ 2.No	☐ 1.Often ☐ 2.Sometimes ☐ 3.Never ☐ 4.Not concerned ☐ 5.Refus
Have you ever punched or hit her with something to hurt her?	☐ 1.Yes → ☐ 2.No	☐ 1.Often ☐ 2.Sometimes ☐ 3.Never ☐ 4.Not concerned ☐ 5.Refus
Have you ever kicked her, dragged her on the floor or ever beaten her ?	☐ 1.Yes → ☐ 2.No	☐ 1.Often ☐ 2.Sometimes ☐ 3.Never ☐ 4.Not concerned ☐ 5.Refus
Have you ever tried to choke or burn her ?	☐ 1.Yes → ☐ 2.No	☐ 1.Often ☐ 2.Sometimes ☐ 3.Never ☐ 4.Not concerned ☐ 5.Refus
Have you ever threatened her with a knife, a gun or another type of weapon?	☐ 1.Yes → ☐ 2.No	☐ 1.Often ☐ 2.Sometimes ☐ 3.Never ☐ 4.Not concerned ☐ 5.Refus
Have you ever physically forced her to have sex with you when she was not willing?	☐ 1.Yes → ☐ 2.No	☐ 1.Often ☐ 2.Sometimes ☐ 3.Never ☐ 4.Not concerned ☐ 5.Refus
Have you ever forced her into carrying out sexual acts she did not want or was against?	☐ 1.Yes → ☐ 2.No	☐ 1.Often ☐ 2.Sometimes ☐ 3.Never ☐ 4.Not concerned ☐ 5.Refus

Q096 - Has it ever happened that your (last) partner beat, boxed, kicked or did something to hurt you physically even though you had never hit or physically attacked her ?

☐ 1.Yes

☐ 2.No

☐ 3.Refus de répondre (ne pas citer)

→ **Passer directement au module H (page 32)**

VERSION ANGLOPHONE

A NE POSER QUE SI LE PATIENT EST UNE FEMME

I will now ask you a few personal questions concerning fatherhood, the desire for a child, and finally relations between couples. I want to remind you that all your responses are confidential and anonymous, and that I am bound by professional secrecy.

Q088_F - Do you have children?

☐ 1.Yes → **How many** ______ ☐ 1.Ne sait pas (ne pas citer) ☐ 2.Refus de répondre (ne pas citer)
☐ 2.No
☐ 3.Refus de répondre (ne pas citer)

Q097 - Do you currently wish or are you trying to have a child ?

☐ 1.Yes
☐ 2.No
☐ 3.Not concerned (post-menopausal woman)
☐ 4.Ne sait pas (ne pas citer)
☐ 5.Refus de répondre (ne pas citer)

Q098 - Have you ever had a miscarriage?

☐ 1.Yes → **Poser la question Q098_1**
☐ 2.No → **Passer à la question Q099**
☐ 3.Refus de répondre (ne pas citer) → **Passer à la question Q099**

Q098_1 - Did this miscarriage necessitate **Une seule réponse possible**

☐ 1.A medical intervention (surgical or invasive)
☐ 2.A non medical intervention (surgical or invasive)
☐ 3.No, the miscarriage/abortion did not need a surgical intervention
☐ 4.Refus de répondre (ne pas citer)

If you will permit me, I will now ask some questions concerning your relations with your current or last partner if you do not have a partner at the moment.

Si la patiente répond spontanément qu'elle n'a jamais eu de partenaire, cocher la case "N'a jamais eu de partenaire" et passer directement au module H (page 32). **N'a jamais eu de partenaire** ☐

Q099 - Who usually makes decisions on how the money you earn is to be spent ?

Ne pas citer les modalités, laissez la personne répondre spontanément

☐ 1.Interviewee/respondent
☐ 2.The Husband/Partner
☐ 3.Interviewee/respondent together with husband/Partner
☐ 4.Someone else
☐ 5.Interviewee/respondent together with somebody else
☐ 6.Other **Specify** ______ **Ecrire en majuscule**
☐ 7.Refus de répondre (ne pas citer)

Q100 - Who usually has the last word in decisions concerning your own health care?

Ne pas citer les modalités, laissez la personne répondre spontanément

☐ 1.Interviewee/respondent
☐ 2.The Husband/Partner
☐ 3.Interviewee/respondent together with husband/Partner
☐ 4.Someone else
☐ 5.Interviewee/respondent together with somebody else
☐ 6.Other **Specify** ______ **Ecrire en majuscule**
☐ 7.Refus de répondre (ne pas citer)

VERSION ANGLOPHONE

Q101 - Has it ever happened that your (previous) partner		Ne pas citer les mentions "Refus" During the past 12 months this has happened :
Says or does something to humiliate you in front of others?	☐ 1.Yes → ☐ 2.No	☐ 1.Often ☐ 2.Sometimes ☐ 3.Never ☐ 4.Not concerned ☐ 5.Refus
Threatens you or someone close to you?	☐ 1.Yes → ☐ 2.No	☐ 1.Often ☐ 2.Sometimes ☐ 3.Never ☐ 4.Not concerned ☐ 5.Refus
Insults you or belittles you?	☐ 1.Yes → ☐ 2.No	☐ 1.Often ☐ 2.Sometimes ☐ 3.Never ☐ 4.Not concerned ☐ 5.Refus

Q102 - Has it ever happened that your (previous) partner does to you any of the following?		Ne pas citer les mentions "Refus" During the past 12 months this has happened :
Hits you, shakes you violently or throws something at you	☐ 1.Yes → ☐ 2.No	☐ 1.Often ☐ 2.Sometimes ☐ 3.Never ☐ 4.Not concerned ☐ 5.Refus
Slaps you ?	☐ 1.Yes → ☐ 2.No	☐ 1.Often ☐ 2.Sometimes ☐ 3.Never ☐ 4.Not concerned ☐ 5.Refus
Twists your arm or pulls your hair?	☐ 1.Yes → ☐ 2.No	☐ 1.Often ☐ 2.Sometimes ☐ 3.Never ☐ 4.Not concerned ☐ 5.Refus
Punches or hits you with something to hurt you?	☐ 1.Yes → ☐ 2.No	☐ 1.Often ☐ 2.Sometimes ☐ 3.Never ☐ 4.Not concerned ☐ 5.Refus
Kicks you, drags you on the floor or fights you?	☐ 1.Yes → ☐ 2.No	☐ 1.Often ☐ 2.Sometimes ☐ 3.Never ☐ 4.Not concerned ☐ 5.Refus
Tries to choke or burn you ?	☐ 1.Yes → ☐ 2.No	☐ 1.Often ☐ 2.Sometimes ☐ 3.Never ☐ 4.Not concerned ☐ 5.Refus
Threatens you with a knife, a gun or any type of arm?	☐ 1.Yes → ☐ 2.No	☐ 1.Often ☐ 2.Sometimes ☐ 3.Never ☐ 4.Not concerned ☐ 5.Refus
Physically forces you to have sex with you even when you don't want to?	☐ 1.Yes → ☐ 2.No	☐ 1.Often ☐ 2.Sometimes ☐ 3.Never ☐ 4.Not concerned ☐ 5.Refus
Forces you to perform other sexual acts even when you don't want to?	☐ 1.Yes → ☐ 2.No	☐ 1.Often ☐ 2.Sometimes ☐ 3.Never ☐ 4.Not concerned ☐ 5.Refus

Q103 - Has it ever happened that you fought, slapped, kicked or did something to physically abuse your (previous) partner even though he has never fought nor abused you physically?

☐ 1.Yes

☐ 2.No

☐ 3.Refus de répondre (ne pas citer)

VERSION ANGLOPHONE

MODULE H: SANTÉ PERÇUE ET QUALITÉ DE VIE

I am now going to ask you questions concerning your general state of heath and the impact the disease has on your daily life.

Q104 - Globally, do you think your health is

☐ 1.Excellent
☐ 2.Very good
☐ 3.Good
☐ 4.Mediocre
☐ 5.Bad
☐ 6.Refus de répondre (ne pas citer)

Q105 - Here is a list of your possible daily activities. For each of them indicate which you can't carry out due to your health

Une réponse par ligne	1. Yes, a lot	2. Yes, a bit	3.No, not at all	(ne pas citer) 4.Refus
Moderate physical activities like sweeping the floor, moving a table, walking for 20 Minutes on a flat field	☐	☐	☐	☐
Climb up many stairs or climb up a very steep slope for a few Minutes	☐	☐	☐	☐

Q106 - Within the past four weeks and due to your physical state

Une réponse par ligne	1. Permanently	2. Very often	3. Often	4. Sometimes	5. Rarely	6. Never	7. Refus
Have you carried out lesser tasks than you had wished?	☐	☐	☐	☐	☐	☐	☐
Have you stopped carrying out certain activities?	☐	☐	☐	☐	☐	☐	☐

Q107 - Within the past four weeks and due to your emotional state (feeling sad, nervous or depressed)

Une réponse par ligne	1. Permanently	2. Very often	3. Often	4. Sometimes	5. Rarely	6. Never	7. Refus
Have you carried out lesser tasks than you had wished?	☐	☐	☐	☐	☐	☐	☐
Have you had difficulties doing what you used to do with as much care and caution?	☐	☐	☐	☐	☐	☐	☐

VERSION ANGLOPHONE

Q108 - During the course of the past four weeks, how much has your physical pain inconvenienced you in your job or in your daily activities?

- ☐ 1.Never
- ☐ 2.Just a bit
- ☐ 3.Moderately
- ☐ 4.A lot
- ☐ 5.Enormously
- ☐ 6.Refus de répondre (ne pas citer)

Q109 - During the course of the past four weeks, are there moments where your physical or emotional state of health hampered your life and your relationship with others (your family, your friends, your acquaintances)?

- ☐ 1.Always
- ☐ 2.Most of the time
- ☐ 3.From time to time
- ☐ 4.Rarely
- ☐ 5.Refus de répondre (ne pas citer)

The following questions concern how you felt during the course of the past four weeks.
(For each question, please indicate the answer which seems the most appropriate).

Q110 - During the course of the past four weeks, are there moments when

Une réponse par ligne	1. Permanently	2. Very often	3. Often	4. Sometimes	5. Rarely	6. Never	(ne pas citer) 7. Refus
You felt calm and relaxed?	☐	☐	☐	☐	☐	☐	☐
You felt overflowing with energy?	☐	☐	☐	☐	☐	☐	☐
You felt sad and defeated?	☐	☐	☐	☐	☐	☐	☐

In the following questions, I am going to ask you about the impact of your seropositivity on your health and life. It is recommended that you reflect on your life during the past two weeks.

Q111 - During the course of the past two weeks, due to your seropositivity

Une réponse par ligne	1. Never	2. Rarely	3.From time to time	4. Often	5. Always	6. Refus
Were you inconvenienced by a change in weight	☐	☐	☐	☐	☐	☐
Were you inconvenienced by skin problem (dry skin, itching, rashes)	☐	☐	☐	☐	☐	☐
Were you inconvenienced by changes in the appearance of some of your body parts (hollowed-out cheek, legs, arms, thinner buttocks, bigger chest or breast, large stomach, accumulation of fats around the neck)	☐	☐	☐	☐	☐	☐
Were you unhappy with the appearance of any part of your body?	☐	☐	☐	☐	☐	☐

MODULE I : LIENS SOCIAUX, DÉVOILEMENT DE LA SÉROPOSITIVITÉ, EXPÉRIENCE DES DISCRIMINATIONS

We will now specifically talk about your experiences with your relatives abour your HIV infection.

Q112 - Since you knew your status have you disclosed it to anybody ?

☐ 1.Yes

☐ 2.No

☐ 3.Refus de répondre (ne pas citer)

→ **Passer directement à la question Q115 (page suivante)**

I will begin by asking you questions on disclosure of your status to your friends and family.

Q113 - For each of the persons i will list out to you, you will tell me if they are aware your HIV status or not. For those who are aware, I will ask you if you told them yourself or not and whether it was accepted or not.

NSP = Ne sait pas (ne pas citer)

If yes

**Does (...........) know you have HIV ?**	1.Not concerned	2.Yes	3.No	4.NSP	**Did you tell them ?** 1.Yes	2.No	**Have they accepted ?** 1.Yes	2.No	3.NSP
Your spouse, or main partner	☐	☐	☐	☐	☐	☐	☐	☐	☐
Your father	☐	☐	☐	☐	☐	☐	☐	☐	☐
Your mother	☐	☐	☐	☐	☐	☐	☐	☐	☐
Brother, Sister	☐	☐	☐	☐	☐	☐	☐	☐	☐
Your child/children	☐	☐	☐	☐	☐	☐	☐	☐	☐
Other members of your family	☐	☐	☐	☐	☐	☐	☐	☐	☐
One or more close friends	☐	☐	☐	☐	☐	☐	☐	☐	☐
A priest, pastor, imam or other religious person	☐	☐	☐	☐	☐	☐	☐	☐	☐
A colleague at work or your employer	☐	☐	☐	☐	☐	☐	☐	☐	☐
Your neighbors	☐	☐	☐	☐	☐	☐	☐	☐	☐

Q114 - Have ever asked someone to help you to dislcose your HIV status?

☐ 1.Yes→ **whom?** ____________________ Ecrire en majuscule

☐ 2.No

☐ 3.Refus de répondre (ne pas citer)

VERSION ANGLOPHONE

In the following questions we will like you to tell us the impact your seropositivity had on your social life.

	1.Yes	2.No	3.Refus de répondre **(ne pas citer)**
**Q115 - Do you have the impression that certain persons are afraid of you because of you are HIV-positive?**	☐	☐	☐
**Q116 - Did you lose any friends after announcing your seropositivity to them ?**	☐	☐	☐
**Q117 - Are there any persons close to you who are afraid of being rejected by others because you are HIV-positive ?**	☐	☐	☐
**Q118 - Do you feel hurt by the way people react when they learn of your HIV status?**	☐	☐	☐
**Q119 - Are there any persons who became more distant to you after hearing of your HIV status ?**	☐	☐	☐
**Q120 - Are there any close persons who stopped calling you when they learnt you had HIV ?**	☐	☐	☐
**Q121 - Are there any persons who showed disgust or recoiled from you due to your status ?**	☐	☐	☐
**Q122 - Are there any persons who act like it was your fault you got infected with HIV ?**	☐	☐	☐
**Q123 - Have you ever experienced any separation from your children and/or family since you learnt of your HIV status ?**	☐	☐	☐
**Q124 - Have you ever lost a job because of your seropositivity?**	☐	☐	☐

Q125 - Can you say you are morally supported

Une réponse par ligne	1. Never	2. A bit	3. Moderately	4. A lot	5. Not concerned	**(ne pas citer)** 6. Refus
By the person you live with as a couple (or your main partner)	☐	☐	☐	☐	☐	☐
By members of your family (Parents, grand parents, brothers, sisters, cousins)	☐	☐	☐	☐	☐	☐
By your acquaintances / friends	☐	☐	☐	☐	☐	☐
By your religious community	☐	☐	☐	☐	☐	☐

VERSION ANGLOPHONE

Q126 - Do you feel lonely ?

☐ 1.Yes
☐ 2.No
☐ 3.Ne sait pas (ne pas citer)
☐ 4.Refus de répondre (ne pas citer)

Q127 - Do you have any acquaintances/friends living with HIV whom you usually visit or go out with?

☐ 1.Yes → **Poser la question Q127_1**
☐ 2.No
☐ 3.Refus de répondre (ne pas citer) → **Passer à la question Q128**

Q127_1 - How many

☐ 1.Just one
☐ 2.From 2 to 5
☐ 3.From 6 to 8
☐ 4.More than 10
☐ 5.Ne sait pas (ne pas citer)
☐ 6.Refus de répondre (ne pas citer)

Q128 - During the past 12 months, have you participated in any activities of associations involved in the fight against AIDS (talk groups, dinners, workshops, etc) ?

☐ 1.Yes
☐ 2.No
☐ 3.Ne sait pas (ne pas citer)
☐ 4.Refus de répondre (ne pas citer)

Noter l'heure de fin de l'entretien

E8 - Interview end of time *(ex: 11:26)* ______
