# Supplementary material for: Intimate partner violence by men living with HIV in Cameroon: Prevalence, associated factors and implications for HIV transmission risk (ANRS-12288 EVOLCAM)
Source: PLoS One. 2021 Feb 18;16(2):e0246192. doi: 10.1371/journal.pone.0246192 (PMC7891744; doi:10.1371/journal.pone.0246192)
Supplement: S1 Table — (DOCX) [file pone.0246192.s003.docx]

**S1 Table. Frequency of 12 perpetrated IPV questionnaire items and detailed results of principal components analysis (PCA) used to construct IPV scores for each form of IPV from these questionnaire items**

| **IPV items** | |  | |  | |  |  | | |  |  |  | **IPV scores** | | | | | | |
| --- | --- | --- | --- | --- | --- | --- | --- | --- | --- | --- | --- | --- | --- | --- | --- | --- | --- | --- | --- |
| *In the past 12 months, have you ever done the following to your female partner:* | | *Never* | | | *Sometimes* | | | | *Often* | | |  | **Psychological** and **physical** IPV score (PPV) | | **Severe physical** IPV score (SPV) | |  | **Sexual** IPV score (SV) | |
| N | % | | N | | | % | n | | % |  | Ranking of IPV items in constructing IPV scores according to weighting from PCA | | | | | | |  |  |
| Said or done something to humiliate her in front of others? | | 351 | 87 | | 38 | | | 9 | 17 | | 4 |  | **0.81*** | 0.2 | | 0.18 | | |  |
| Insulted or belittled her? | | 351 | 86 | | 38 | | | 9 | 17 | | 4 |  | **0.83*** | 0.29 | | 0.22 | | |  |
| Threatened or someone close to her? | | 327 | 81 | | 53 | | | 13 | 26 | | 6 |  | **0.82*** | 0.21 | | 0.29 | | |  |
| shaken forcefully or thrown something at her? | | 362 | 89 | | 31 | | | 7 | 13 | | 3 |  | **0.7*** | **0.67*** | | 0.23 | | |  |
| Slapped her? | | 351 | 86 | | 38 | | | 9 | 17 | | 4 |  | **0.63*** | **0.61*** | | 0.29 | | |  |
| Twisted her arm or pulled her hair? | | 388 | 96 | | 15 | | | 4 | 3 | | 1 |  | 0.38 | **0.69*** | | -0.02 | | |  |
| punched or hit her with something to hurt her?? | | 400 | 99 | | 6 | | | 1 | 0 | | 0 |  | 0.07 | **0.72*** | | -0.06 | | |  |
| Kicked her, dragged her on the floor or beaten her? | | 399 | 98 | | 5 | | | 1 | 2 | | 0 |  | 0.15 | **0.57*** | | 0.12 | | |  |
| Threatened her with a gun, knife, or another type of weapon? | | 406 | 100 | | 0 | | | 0 | 0 | | 0 |  | NA | NA | | NA | | |  |
| Tried to choke or burn her ? | | 406 | 100 | | 0 | | | 0 | 0 | | 0 |  | NA | NA | | NA | | |  |
| Physically forced her to have sex even when she was not willing? | | 396 | 98 | | 6 | | | 1 | 4 | | 1 |  | 0.2 | 0.06 | | **0.87*** | | |  |
| Forced her into carrying out sexual acts she did not want or was against? | | 402 | 99 | | 1 | | | 0 | 3 | | 1 |  | 0.29 | 0.06 | | **0.83*** | | |  |
|  | |  | |  | |  | Eigen values | | | | |  | 3.2 | 2.3 | | 1.8 | | |  |
|  | |  | |  | |  | Explained variance (%) | | | | |  | 32 | 23 | | 18 | | |  |
|  | | Cumulative variance (%) | | | | | | | | | |  |  | 73 | | | | |  |
|  | |  | |  | | Cronbach’s α | | | | | |  | 0.83 | 0.69 | | 0.62 | | |  |
|  | *Correlation with other IPV scores* | | | | | | | | | | |  |  |  | |  | | |  |
|  | | Psychological and physical IPV score | | | | | | | | | |  | - | 0.36 | | 0.29 | | |  |
|  | | Severe physical IPV score | | | | | | | | | |  | - | - | | 0.08 | | |  |
|  | | Sexual IPV score | | | | | | | | | |  | - | - | | - | | |  |

*Bold values indicate that the IPV item strongly contributed to the construction of the IPV score (weighting >0.5)
